# Structural Design of PES-CS-MMT Composite Membrane by Layer-by-Layer Self-Assembly for the Removal of Antibiotic Wastewater

**DOI:** 10.3390/membranes16050180

**Published:** 2026-05-20

**Authors:** Zhiyuan Shi, Xinhao Sun, Jiayi Ren, Weixiang Xu, Qianshuo Guo, Yinxi Chen, Zhengda Lin, Yu Tian, Jun Zhang

**Affiliations:** National Engineering Research Center for Safe Disposal and Resources Recovery of Sludge, School of Environment, Harbin Institute of Technology, Harbin 150090, China

**Keywords:** layer-by-layer assembly, multilayer gradient membrane, composite membrane, antibiotic removal, wastewater treatment

## Abstract

**Highlights:**

1. LbL assembly of CS and MMT creates a tunable gradient multi-layer membrane.
2. Synergistic organic-inorganic layers enhance thermal and structural stability.
3. Gradient design balances interfacial adsorption with rapid mass transfer channels.
4. Distinct kinetic selectivity among Diclofenac revealed via dynamic testing.
5. Provides structural basis for controllable antibiotic-selective membrane design.

**Abstract:**

A multilayer gradient composite membrane was fabricated on a PES ultrafiltration substrate through layer-by-layer assembly of chitosan (CS) and montmorillonite (MMT), followed by Ca^2+^ crosslinking. The designed architecture forms a multi-layer gradient composite membrane through successive self-assembly, aiming to balance adsorption, interfacial transport and structural stability. SEM observations showed a clear stratified configuration with relatively uniform thickness distribution, including a relatively dense MMT-rich surface layer and a porous PES support that preserved mass-transfer channels. FTIR confirmed the introduction of hydroxyl/amino-containing CS and aluminosilicate-related MMT species onto the membrane surface, indicating successful incorporation of both organic and inorganic components. TG–DTG results further suggested enhanced thermal stability arising from the cooperative effect of the inorganic lamellae and the polymer framework. In dynamic tests, the membrane displayed concentration-responsive adsorption behavior toward gatifloxacin, ciprofloxacin and ofloxacin, and different pollutants reached equilibrium or quasi-steady states at different rates. Comparative kinetic results at the same initial concentration showed that diclofenac, gatifloxacin and ciprofloxacin approached stable plateaus much faster, whereas ofloxacin increased slowly and did not reach an obvious plateau within the tested period. These results indicate that pollutant removal was jointly governed by interfacial interactions, gradient-layer diffusion resistance and overall transport behavior rather than by concentration alone. Overall, the layer-by-layer strategy provided a controllable route for constructing gradient functional layers on PES membranes, demonstrating potential for advanced treatment of antibiotic-containing wastewater and related pharmaceutical effluents.

## 1. Introduction

Against the backdrop of the extensive application of antibiotics in medical treatment, livestock and poultry breeding, and aquaculture. Their residues are continuously entering various water environment media such as municipal sewage and breeding drainage [1,2]. Because some antibiotics cannot be completely metabolized by the body after use and retain their biological activity. When they enter the sewage system, they not only pose a new risk of organic pollution, but also may promote the enrichment and spread of drug-resistant bacteria and drug-resistant genes through selective pressure [3,4]. The World Health Organization has pointed out that antimicrobial compounds, drug-resistant microorganisms and their genes have become common pollutants in wastewater and sludge [5].

Meanwhile, the treatment of antibiotic wastewater still faces multiple challenges. Firstly, antibiotics come in a wide variety, including tetracyclines, fluoroquinolones, etc. Different molecules have significant differences in charge characteristics, hydrophobicity, and molecular size, which leads to highly complex migration, transformation behaviors and removal mechanisms in water [6]. Yanyan Jia et al. [7] summarized the mechanisms by which biochar removes antibiotics, including non-biological adsorption such as electrostatic interaction and hydrogen bonding, as well as biological synergistic effects such as biochar acting as an electron mediator. They also analyzed the influence of raw materials, pyrolysis conditions, and modification methods on the removal efficiency, and discussed the prospects and challenges of engineering applications. Secondly, real wastewater systems are usually accompanied by natural organic matter, suspended particles, inorganic salts, and coexisting organic pollutants. These components will compete with antibiotics for adsorption, complexation or interface shielding, thereby reducing the stability and selectivity of a single treatment unit [8]. Jian Yang et al. [9] synthesized a magnetic biochar/bismuth tungstate composite catalyst for the removal of ciprofloxacin (CIP) and tetracycline (TC) from water. In a dual-solute system containing both ciprofloxacin (CIP) and tetracycline (TC), the catalyst showed preferential adsorption selectivity toward CIP, with competitive adsorption occurring between the two antibiotics. Coexisting anions could impair the efficiency of the integrated adsorption-photocatalysis removal process by scavenging reactive free radicals or blocking active sites on the catalyst surface. Finally, the design goals of conventional wastewater treatment systems mainly focus on reducing suspended solids, organic matter and nutrients, rather than precisely controlling trace antibiotics and their associated resistance risks. Therefore, the treated effluent may still be an important anthropogenic source for the release of antibiotic residues, resistant bacteria and resistant genes into the environment [10]. Rumeng Wang et al. [11] conducted an investigation on antibiotics and antibiotic resistance genes (ARGs) in the secondary effluent of wastewater treatment plants (WWTP) and the receiving water bodies. In relatively closed lakes, the effluent significantly influenced the distribution of antibiotics and ARGs. In the treatment of reclaimed water, units such as chlorination and ozonation sometimes led to an increase in their content.

In response to the aforementioned issues, various types of antibiotic wastewater treatment approaches have been developed both domestically and internationally, mainly including biological treatment, adsorption methods, advanced oxidation processes, as well as membrane separation coupled with other technologies [12,13,14,15]. Biological methods have the advantages of relatively low operating costs and suitability for reducing conventional organic loads. However, they often have limited degradation capabilities for antibiotics with stable structures, high toxicity, or significant antibacterial effects [16]. Huimin Wei et al. [17] demonstrated that the aquatic plant Hydrilla verticillata can, by regulating the microbial community, synergistically remove antibiotics and inhibit the spread of resistance genes. The adsorption method is regarded as a practical technical approach for removing peak concentration drug contamination due to its simple process, quick startup, and good buffering capacity for shock loads [18]. Kangying Guo et al. [19] employed the coagulation method, using a dual coagulation process consisting of Polyaluminum chloride (PAC) and Poly dimethyl diallyl ammonium chloride (PDMDAAC), to remove the composite pollutants of chlortetracycline (CTC) and humic acid (HA). Advanced oxidation technologies rely on hydroxyl radicals, sulfate radicals or photocatalytic active species to break the chains, transform or even mineralize antibiotic molecules. In recent years, they have received extensive attention in the deep treatment of pharmaceutical wastewater [20,21]. Weijie Song et al. [22] summarized the research progress of advanced oxidation processes using persulfate for the removal of antibiotic resistance genes (ARGs) and resistant bacteria (ARBs). Both homogeneous catalysis and heterogeneous catalysis can generate reactive oxygen species, which effectively degrade ARGs and inactivate ARB through free radical and non-free radical pathways, providing strategies for controlling environmental antibiotic resistance. Membrane technology, through multiple mechanisms such as size sieving, charge repulsion and interface adsorption, can achieve efficient separation within a relatively short residence time. It can also be coupled with adsorption, catalysis and other functional units to form a more efficient composite purification system [23,24,25]. Xiaoyue Yao et al. [26] developed a novel electrocatalytic composite membrane (PES/CP/CoCo-PBA), which, through electrochemical activation of persulfate (PMS), provides abundant active sites on the membrane surface with CoCo-PBA nano-catalysts. This membrane also exhibits excellent anti-pollution performance and broad-spectrum antibiotic removal capability.

Although the existing methods have their own advantages, there are still significant bottlenecks in their engineering applications. The simple biological method is often limited by the inhibitory effect of antibiotics and the retention time, making it difficult to achieve stable and deep removal [27]. The adsorption method essentially involves a phase transfer process of pollutants. The recovery, regeneration and safe disposal of saturated adsorbents remain significant environmental and economic challenges [28]. Although the advanced oxidation process has a high removal efficiency, the environmental risks of the drug conversion products and by-products cannot be ignored. Moreover, the chemical reagents or energy consumption required are relatively high [29]. The membrane principle generally encounters problems such as flux attenuation, concentration polarization and membrane fouling. Especially in real antibiotic wastewater, the coexistence of organic substances and colloidal particles is more likely to cause surface contamination and pore blockage [30]. In addition, traditional single-layer functional coating membranes often encounter problems such as uneven distribution of the active layer, difficult control of thickness, mutual constraints between selectivity and flux, and insufficient long-term operational stability [31]. Therefore, how to establish a more reasonable balance between “efficient removal—low mass transfer resistance—anti-pollution—renewable” through structural design has become the key issue in current research on membrane treatment of antibiotic wastewater.

To address these technical bottlenecks, this study employs a layer-by-layer (LbL) self-assembly strategy to immobilize chitosan (CS) and montmorillonite (MMT) onto the surface of a polyethersulfone (PES) ultrafiltration membrane, followed by Ca^2+^ cross-linking to fabricate a multilayer composite membrane with well-defined compositional and functional gradients. The scientific basis of this approach lies in the fact that chitosan is rich in amino and hydroxyl groups, which can provide electrostatic adsorption, hydrogen bond binding, and interface hydrophilic regulation sites [32]. Montmorillonite possesses a layered nanosheet structure, a high specific surface area, and good chemical stability, which can enhance the rigidity, interface confinement, and molecular sieving ability of the membrane layer [33]. The self-assembly process of layers is conducive to the controllable adjustment of the thickness, surface chemistry, and interlayer construction sequence of the membrane layer under mild conditions. Thereby avoiding the accumulation of defects and the imbalance of mass transfer caused by a single coating [34]. This study will explore the performance of multi-layer gradient composite membranes in terms of representative antibiotic removal, continuous operation, anti-pollution and regeneration stability through a stepwise self-assembly gradient configuration. It will further reveal the correlation mechanism among the structure, interface and separation properties. The aim of this paper is to provide a membrane material solution with both designability and verifiability for the deep treatment of antibiotic wastewater, and to offer new experimental basis and structural design ideas for the environmental performance evaluation, material optimization and engineering application of related treatment technologies.

## 2. Material and Method

### 2.1. Chemicals and Materials

The chemicals and materials used in this experiment are as follows. A commercial PES ultrafiltration membrane with a molecular weight cut-off of 30 kDa and a diameter of 47 mm was selected as the base membrane [35,36]. Using an analytical-grade chitosan (CS) with a deacetylation degree of no less than 90% as the organic phase material, it is used to provide adsorption sites [37,38]. Using an analytical-grade sodium-based montmorillonite (MMT) with a particle size of 50–100 nm as the inorganic phase material to enhance the adsorption performance and mechanical strength [39,40]. Analytical-grade calcium chloride (CaCl_2_) was selected as the cross-linking agent to strengthen the binding between CS and MMT [41]. The pH value of the system was adjusted by using pure hydrochloric acid (HCl) and sodium hydroxide (NaOH). Deionized water was prepared in the laboratory and was used as the solvent and cleaning solution. The relevant instruments and equipment are as follows. The HH-S6 type constant temperature water bath pot was used to control the assembly temperature. The H-1850 type desktop high-speed centrifuge was used to remove the un-separated particles in the dispersion liquid. The CMT6104 type universal testing machine was used to test mechanical properties such as tensile strength, and the homemade counter-flow filtration device with an effective area of 50 cm^2^ was used to conduct dynamic filtration and collaborative removal experiments. The Agilent 1260 type high-performance liquid chromatography (HPLC) was used to detect antibiotic concentrations.

### 2.2. Membrane Preparation

**Figure 1:**
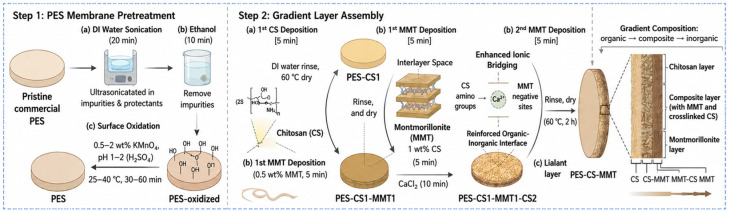
Schematic diagram of the synthesis mechanism of PES-CS-MMT gradient membrane.

In this study, a PES-based substrate was used as the base template, and the PES-CS-MMT gradient membrane was constructed by the layer-by-layer self-assembly method. The synthesis mechanism is shown in Figure 1. The pretreatment of the PES base membrane involves preliminary cleaning and plasma surface modification. First, the PES membrane is cut into circular discs with a diameter of 50 mm. It is then ultrasonically cleaned with deionized water for 30 min (power 200 W), followed by ultrasonically cleaning with anhydrous ethanol for 15 min to remove surface protectants and organic impurities. The membrane is then rinsed multiple times with deionized water and is ready for use. Subsequently, the cleaned membrane is placed flat on the sample stage of the plasma treatment instrument, evacuated to a pressure of ≤10 Pa, and oxygen with a purity of 99.99% (flow rate 10 sccm, maintaining chamber pressure 30 Pa) is introduced. The treatment is carried out at a power of 100 W for 30 s (the optimized parameter range is power 80–120 W, time 20–40 s). After the treatment, the membrane is naturally cooled to room temperature and labeled as PES-plasma. It is used in subsequent experiments within 48 h to avoid oxidation of surface functional groups.

Subsequently, the composite membrane was prepared. First, a 2 wt% chitosan solution was prepared. 2.0 g of chitosan was dissolved in 100 mL of 2% (*v*/*v*) dilute acetic acid, and magnetic stirring was carried out for about 5 h until complete dissolution. The solution was filtered through a 0.22 μm filter membrane to remove impurities. At the same time, a 1 wt% montmorillonite dispersion solution was prepared. 1 g of montmorillonite was dispersed in 100 mL of deionized water, and ultrasonic treatment at 300 W for 1 h was performed to cause it to disintegrate and form a stable colloid. After 1 h of standing, the supernatant was collected. Another 0.5 wt% CaCl_2_ cross-linking solution (0.5 g/100 mL deionized water) was prepared for later use. Using the modified PES oxide membrane as the base, it was first immersed in a 2 wt% chitosan solution and left at 25 °C for 30 min to allow the chitosan to anchor onto the membrane surface through electrostatic and hydrogen bond interactions. The membrane was then rinsed with deionized water and dried at 60 °C for 1 h. Subsequently, the membrane was immersed in a 1 wt% montmorillonite dispersion solution for 10 min to allow the negatively charged montmorillonite layers to adsorb onto the positively charged chitosan layer. After rinsing, it was dried at 60 °C for 1 h. Then, it was immersed in a 2 wt% chitosan solution for about 3 min, rinsed and immediately immersed in a 0.5 wt% CaCl_2_ solution for 10 min to achieve ionic bridging between the chitosan chains and the montmorillonite layer. It was then dried at 60 °C for 1 h. Finally, it was immersed in a 1 wt% montmorillonite dispersion solution for 15 min, rinsed, and dried at 60 °C for 2 h, resulting in a layered self-assembled gradient structure, which is collectively referred to as the PES-CS-MMT composite membrane.

### 2.3. Performance Test

**Figure 2:**
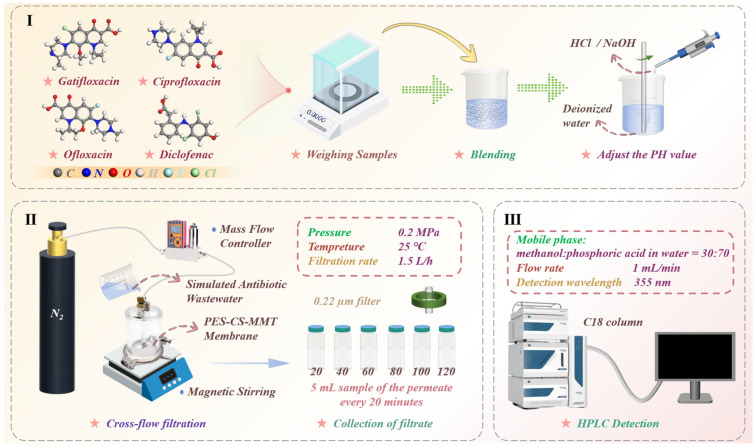
Schematic diagram of the performance testing process of PES-CS-MMT composite membrane.

A variety of typical antibiotics were selected for performance testing of the PES-CS-MMT composite membrane. The schematic diagram of the entire experimental process is shown in Figure 2.

#### 2.3.1. Cross-Flow Filtration Setup and Operating Conditions

In the cross-flow filtration test, the prepared composite membrane (effective area A = 50 cm^2^ = 0.005 m^2^) was fixed in the membrane module. Under an operating transmembrane pressure (ΔP) of 0.2 MPa (2 bar), the cross-flow velocity was set at 1.5 L/h. All experiments were conducted at room temperature (25 ± 1 °C).

#### 2.3.2. Pure Water Permeability Measurement

A blank experiment using pure water was conducted first as a control. The system was continuously operated for 15 min, and the pure water permeability (P, $L\cdot m^{-2}\cdot h^{-1}\cdot bar^{-1}$) was recorded and calculated using Equation (1) [42]:

(1) $$P=\frac{V}{A\cdot \Delta t\cdot \Delta P}$$

where V is the volume of the permeate (L) and Δt is the permeation time (h).

#### 2.3.3. Removal Efficiency and Rejection Rate Calculation

Subsequently, simulated antibiotic wastewater of different initial concentrations (*C_f_*, mg/L) was introduced. The permeate concentration (*C_p_*, mg/L) was determined by HPLC. Two distinct removal metrics were defined to distinguish between transient adsorption-dominated removal and steady-state intrinsic separation performance:

(1) Instantaneous total removal efficiency (*A_total_*, %): Represents the combined effect of membrane adsorption and size/charge rejection at any time during the initial filtration stage, calculated as: [43]

(2) $$A_{total}=\left(1-\frac{C_{p,i}}{C_{f}}\right)\times 100\%$$

where $C_{p,i}$ is the antibiotic concentration in the i-th permeate sample.

(2) Steady-state rejection rate (*R*, %): Reflects the intrinsic separation capability of the membrane after adsorption equilibrium is reached (when permeate concentration stabilizes for 3 consecutive sampling points), calculated as follows [43]:

(3) $$R=\left(1-\frac{C_{p,steady}}{C_{f}}\right)\times 100\%$$

where $C_{p,steady}$ is the average permeate concentration at steady state.

#### 2.3.4. Dynamic Adsorption Capacity Calculation

To accurately evaluate the dynamic adsorption performance of the membrane, the normalized dynamic adsorption capacity (*q_t_*, mg/m^2^) at filtration time t was calculated using Equation (4):

(4) $$q_{t}=\frac{(C_{f}-C_{p,i})\cdot Q\cdot t}{A}$$

where Q is the volumetric flow rate of the permeate (L/h), t is the filtration time (h), and A = 0.005 m^2^ is the effective membrane area.

#### 2.3.5. Anti-Fouling Performance Test

To investigate the anti-pollution performance of the composite membrane, the system was continuously operated for 72 h, and the flux attenuation rate ($\eta_{1}$, %) was calculated using Equation (5):

(5) $$\eta_{1}=\left(1-\frac{J_{steady}}{J_{0}}\right)\times 100\%$$

where $J_{steady}$ is the average steady-state flux of antibiotic solution, and $J_{0}$ is the initial pure water flux.

In addition, the reversible fouling ratio ($R_{r}$, %) and irreversible fouling ratio ($R_{ir}$, %) were calculated to analyze the fouling mechanism:

(6) $$R_{r}=\left(1-\frac{J_{hydraulic}}{J_{0}}\right)\times 100\%$$

(7) $$R_{ir}=\left(1-\frac{J_{chemical}}{J_{0}}\right)\times 100\%$$

where $J_{hydraulic}$ is the pure water flux after hydraulic cleaning (rinsing with deionized water for 30 min), and $J_{chemical}$ is the pure water flux after chemical cleaning (rinsing with dilute HCl solution pH = 2 for 10 min followed by deionized water).

#### 2.3.6. Regeneration and Reusability Test

In the recyclability experiment, the used membrane was subjected to 5 min of ultrasonic treatment, rinsed with dilute HCl solution (pH = 2) 3–5 times, followed by multiple rinses with deionized water. The washed membrane was dried at 70 °C for 2 h, and the flux recovery rate ($\eta_{2}$, %) was calculated using Equation (8):

(8) $$\eta_{2}=\left(\frac{J_{regeneration}}{J_{0}}\right)\times 100\%$$

where $J_{regeneration}$ is the pure water flux of the cleaned membrane.

All performance tests were performed in triplicate (*n* = 3) using independently prepared membrane coupons. All numerical data are presented as mean ± standard deviation (SD). Statistical significance was analyzed using one-way ANOVA, and differences with *p* < 0.05 were considered statistically significant.

#### 2.3.7. Complex Matrix Interference Test

To evaluate the membrane’s performance under real wastewater conditions, three types of interference experiments were conducted:

(1) NOM interference test: Humic acid (HA) was added to the 300 mg/L antibiotic solution at concentrations of 10, 20, 30, 40, and 50 mg/L
(2) Inorganic salt interference test: NaCl (0.1, 0.3, 0.5 M), CaCl_2_ (0.01, 0.05, 0.1 M), and MgSO_4_ (0.01, 0.05, 0.1 M) were added separately to the 300 mg/L antibiotic solution
(3) Mixed antibiotics test: A mixed solution containing equal concentrations of diclofenac, gatifloxacin, ciprofloxacin, and ofloxacin was prepared with a total concentration of 300 mg/L

All interference tests were conducted under the same operating conditions as the single-solute experiments (0.2 MPa, 1.5 L/h cross-flow velocity).

### 2.4. Characterization Methods

This study employed a variety of characterization methods to analyze the surface properties of the membrane. The surface morphology of the membrane was observed using a scanning electron microscope (SEM), and the contents of C, O, F, Ti, and Si elements on the membrane surface were determined using an energy dispersive X-ray spectrometer attached to it. The mass changes of the sample during the heating process were measured by thermogravimetric analysis-differential thermal analysis (TG-DTG) to evaluate its thermal stability, composition, and decomposition behavior. Fourier transform infrared spectroscopy (FTIR) was used to verify the modification process, and atomic force microscopy (AFM) was used to test the morphology and physical and chemical properties of the original PES membrane and the modified PES membrane. Surface wettability was measured by the sessile drop method using a contact angle goniometer. The surface zeta potentials of the membranes were determined by a streaming potential analyzer to characterize the surface charge characteristics. The mechanical properties of the membranes were tested using a universal testing machine. Post-adsorption characterizations including contact angle measurement, cross-sectional SEM observation and AFM tapping-phase analysis were performed to evaluate the structural stability and adsorption uniformity of the composite membrane after antibiotic adsorption.

## 3. Result and Discussion

### 3.1. Characterization Results

#### 3.1.1. SEM Analysis of PES-CS-MMT Composite Membrane

**Figure 3:**
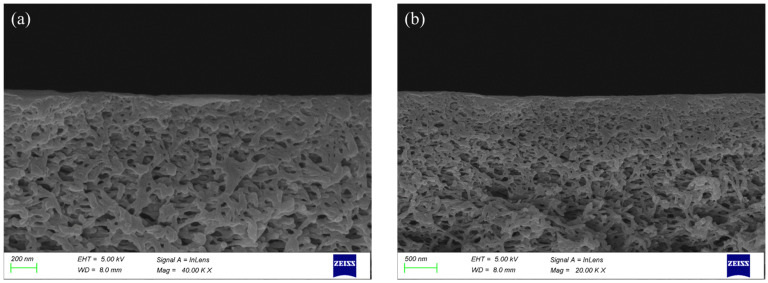
Two scanning electron microscope images of the cross-section of the PES-CS-MMT composite membrane at two different magnifications. (**a**) 40.00k× magnification; (**b**) 20.00k× magnification.

The SEM images of the membrane cross-section are shown in Figure 3a,b. The thickness distribution of each cycle layer of the multi-layer composite membrane is relatively uniform, indicating that its overall structure has good uniformity. At the same time, the images also initially present the basic layered configuration of the composite membrane. Specifically, in the preparation process of the PES-CS-MMT membrane, the chitosan and montmorillonite were successively deposited on the surface of the base membrane by the self-assembly method layer by layer, ultimately forming a multi-layer composite structure with gradient characteristics.

**Figure 4:**
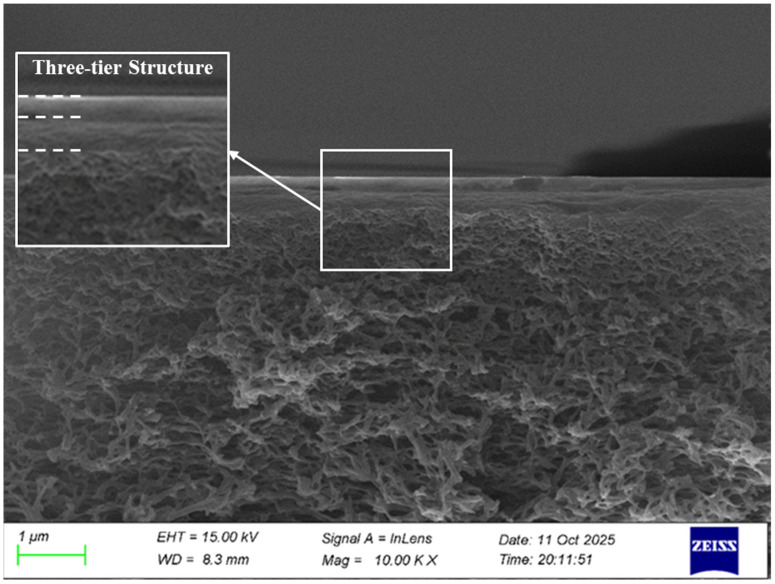
The three-layer structure of PES-CS-MMT. (The dashed lines in the text indicate and divide the three different structural levels of the composite membrane’s cross-section).

Figure 4 clearly presents this three-layer structure. The outer layer is the montmorillonite (MMT) layer, with a thickness of approximately 1 μm, and the pore sizes are mostly in the range of several tens to several hundred nanometers. It is relatively dense, and the pore sizes are smaller and more densely distributed. The middle layer is the chitosan (CS) layer, with pore sizes slightly larger than those of the MMT layer. The pore connectivity of each layer is good, and together they form the CS-MMT composite functional layer. Generally, it is believed that this composite layer not only avoids the blocking of the membrane pores by direct coverage of the functional layer but also, through precise layered structure design, will have better retention performance. The bottom layer is the PES base membrane, with a thickness occupying most of the cross-section, and the size is mostly in the range of several hundred nanometers to 1 μm. It is mostly an asymmetric porous structure, with uniform pore diameters, and an internal sponge-like or finger-like pore channel. This structure enables the PES base membrane to provide strong mechanical support and basic permeability, ensuring the physical toughness of the membrane and a permeation capacity far higher than that of ordinary water-permeable membranes, which is conducive to mass transfer.

In subsequent tests, through the analysis of subsequent performance test data, if it is shown that the collaborative removal efficiency of the two is better than the individual removal efficiency, it can be inferred that antibiotics and heavy metal ions have a mutual promoting effect in membrane removal. Then, by analyzing the particles retained on the membrane surface through nuclear magnetic resonance and spectrophotometry, to determine the molecular structure and chemical composition of the particles, and thereby comprehensively analyze the specific mechanism of PES-CS-MMT retaining antibiotics/heavy metal ions.

#### 3.1.2. TG-DTG Analysis of the PES-CS-MMT Composite Membrane

**Figure 5:**
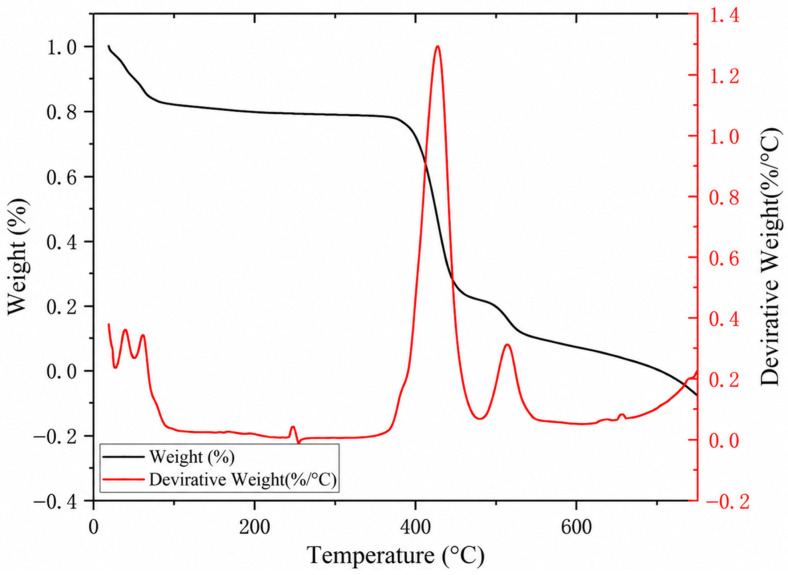
TG-DTG curves of the PES-CS-MMT composite membrane.

From the corresponding relationship between the mass retention curve and the weight loss rate curve in Figure 5, it can be seen that the PES-CS-MMT composite membrane does not undergo instability in a single step during the heating process, but rather undergoes a continuous evolution from slow change to rapid decomposition and subsequent attenuation. From room temperature to approximately 120 °C, only a limited decrease in mass occurs, which is more consistent with the release of adsorbed water and a small amount of weakly bound small molecules. The curve in the subsequent wider temperature range is relatively flat, indicating that the main structure of the membrane body can maintain good integrity at medium and low temperatures. When the temperature approaches 372 °C, the weight loss begins to significantly accelerate, and a peak decomposition rate signal appears near 426.78 °C. A weaker subsequent thermal event can be identified at 513.65 °C, indicating that there is still further structural decomposition or residual organic phase evolution at high temperatures.

From the perspective of structural design, this thermal response feature can support the synergistic stabilizing mechanism of “surface rich in inorganic layers, internal bearing by polymer framework” formed by layer-by-layer self-assembly. Compared with the common earlier main degradation interval of chitosan, the key decomposition process of this composite membrane is overall shifted backward, indicating that the MMT enriched on the outer layer effectively inhibits heat transfer and the diffusion of decomposition products outward, while the aromatic sulphone main chain of PES continues to provide framework support, preventing the membrane body from losing structural constraints rapidly at lower temperatures. In conclusion, the MMT surface layer does not act alone but works together with the PES support network to enhance the interface constraint and overall stability under thermal disturbances, which is particularly important for scenarios involving hot water, chemical cleaning or regeneration treatment of antibiotic wastewater separation.

#### 3.1.3. Comparative Analysis of FTIR Spectra of PES Membrane and PES-CS-MMT Composite Membrane

**Figure 6:**
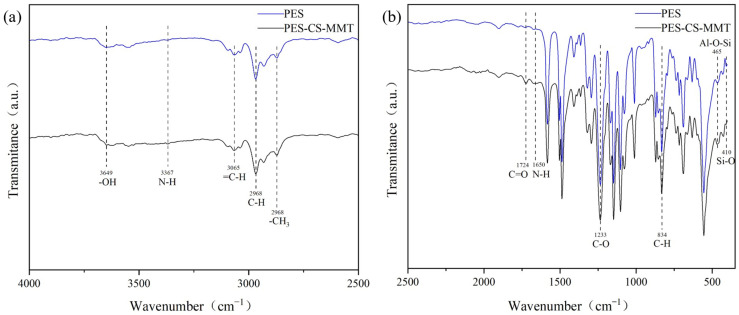
FTIR spectra of pristine PES membrane and PES-CS-MMT composite membrane: (**a**) 4000–2500 cm^−1^ functional group region; (**b**) 2500–400 cm^−1^ fingerprint region.

For the FTIR spectroscopic analysis, Figure 6 shows the FTIR spectra of the pristine PES membrane and the PES-CS-MMT composite membrane, and the spectra were divided into two regions for detailed interpretation.

(1) 4000–2500 cm^−1^ functional group region (Figure 6a): The PES-CS-MMT composite membrane exhibits significantly enhanced absorption in this region compared to the pristine PES membrane. A broad and strong absorption band centered at 3432 cm^−1^ is observed in the composite membrane, which is attributed to the stretching vibrations of O-H and N-H groups from chitosan. This confirms the successful introduction of hydroxyl and amino-containing chitosan onto the membrane surface. Additionally, a weak absorption peak at 2968 cm^−1^ corresponding to aliphatic C-H stretching vibrations appears in the composite membrane, which is absent in the pure PES membrane, further verifying the presence of chitosan.
(2) 2500–400 cm^−1^ fingerprint region (Figure 6b): Several new characteristic peaks are clearly visible in the composite membrane in this region. The peaks at 1650 cm^−1^ and 1560 cm^−1^ are assigned to the amide I and amide II vibrations of chitosan, respectively. The absorption peak at 1233 cm^−1^ in the composite membrane shows a slight shift and intensity increase compared to the PES membrane, which is due to the superposition of C-O stretching vibrations from chitosan and the intrinsic sulfone group vibrations of PES. Most importantly, two distinct peaks at 465 cm^−1^ and 410 cm^−1^ appear in the composite membrane, which correspond to the Si-O-Si and Al-O-Si bending vibrations of the montmorillonite aluminosilicate skeleton. These peaks provide direct evidence for the successful incorporation of MMT into the composite membrane.

By combining the characteristic peaks from both spectral regions, it can be reliably concluded that chitosan and montmorillonite have been successfully assembled onto the PES base membrane through layer-by-layer self-assembly, and stable interfacial interactions (hydrogen bonds and electrostatic attractions) have been formed between the layers.

#### 3.1.4. Contact Angle Analysis

**Figure 7:**
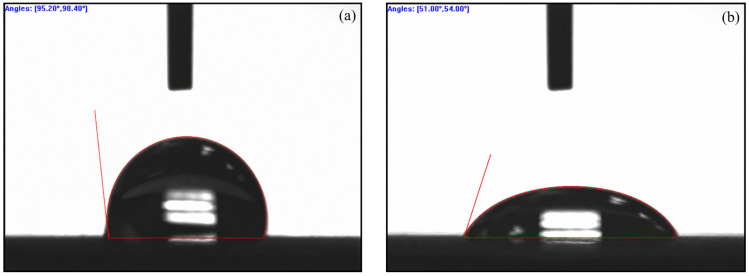
Water contact angle images of the pristine PES membrane and the PES-CS-MMT composite membrane. (**a**) Pristine PES membrane; (**b**) PES-CS-MMT composite membrane. Note: The measured contact angle values are labeled in the upper-left corner of each image. (The red line is used to visually display the defined boundary and measurement reference of the contact angle).

Surface wettability, as quantified by water contact angle, is a fundamental membrane property governing adsorption affinity for polar pollutants and anti-fouling behavior. The contact angle measurements for the pristine PES membrane and the PES-CS-MMT composite membrane are presented in Figure 7.

For the pristine PES membrane (Figure 7a), two parallel measurements yield contact angles of 95.20° and 98.40°, corresponding to an average value of 96.8 ± 1.6°. This high contact angle is consistent with the inherent hydrophobicity of commercial PES ultrafiltration membranes, which arises from the non-polar aromatic backbone of the PES polymer. The lack of polar functional groups on the membrane surface results in weak water affinity, forming a spherical water droplet with a large contact angle.

In sharp contrast, the PES-CS-MMT composite membrane (Figure 7b) exhibits dramatically improved hydrophilicity, with measured contact angles of 51.00° and 54.00° and an average of 52.5 ± 1.5°. The significant reduction in contact angle (by ~44°) directly confirms the successful introduction of polar functional groups via layer-by-layer assembly of chitosan (CS) and montmorillonite (MMT). The abundant hydroxyl (-OH) and amino (-NH_2_) groups in CS, combined with the silanol (Si-OH) and aluminol (Al-OH) groups on the outermost MMT layer, provide numerous sites for hydrogen bonding with water molecules, thereby enhancing surface wettability. Notably, the surface properties of the composite membrane are dominated by the outermost MMT layer, whose high density of hydrophilic hydroxyl groups is the primary contributor to the enhanced hydrophilicity.

This improved hydrophilicity is highly beneficial for the membrane’s practical application: it enhances the membrane’s affinity for polar antibiotic molecules, facilitating the adsorption process; simultaneously, it helps form a hydration layer on the membrane surface, mitigating irreversible fouling during cross-flow filtration, as supported by the flux stability results in subsequent sections.

#### 3.1.5. AFM Analysis of the PES-CS-MMT Composite Membrane

**Figure 8:**
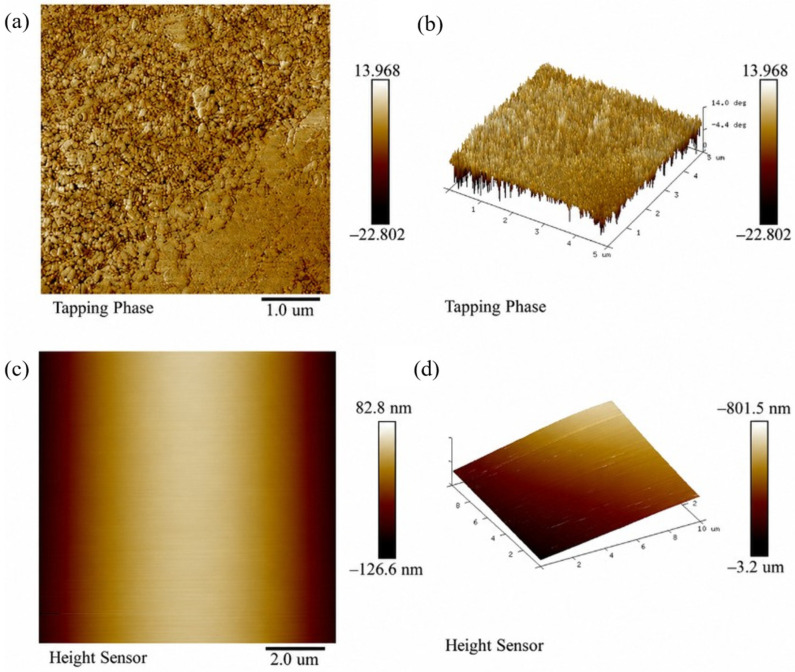
AFM topographic images of the pristine PES membrane and PES-CS-MMT composite membrane. (**a**) 2D tapping-phase image of the PES-CS-MMT composite membrane; (**b**) 3D tapping-phase image of the PES-CS-MMT composite membrane; (**c**) 2D height sensor image of the pristine PES membrane; (**d**) 3D height sensor image of the pristine PES membrane.

Figure 8 displays the AFM surface topography of both the pristine PES membrane and the PES-CS-MMT composite membrane. For the pristine PES membrane (Figure 8c,d), height-mode imaging reveals an inherently smooth and uniform surface with minimal undulations. The arithmetic mean roughness (R_a_) and root-mean-square roughness (R_q_) are only 30.4 nm and 35.2 nm, respectively, consistent with the typical low roughness of commercial ultrafiltration PES membranes. No distinct protrusions or compositional heterogeneities are observed, confirming the homogeneous nature of the base membrane.

In sharp contrast, the PES-CS-MMT composite membrane (Figure 8a,b) exhibits drastically altered surface characteristics under tapping-phase imaging (sensitive to surface composition and mechanical property variations). The average phase shift of 3.38° (within the 5 μm scan range) falls into the “extremely high phase heterogeneity” category, indicating high-frequency and significant variations in local material properties across the membrane surface. The phase fluctuation of 0.68° per micrometer further confirms the formation of a heterogeneous surface, directly arising from the successful deposition of chitosan and montmorillonite layers via layer-by-layer assembly. This pronounced phase variation corresponds to the dense, granular morphology observed in the 2D and 3D phase images, generating abundant three-dimensional adsorption sites. These structural features enable antibiotic molecules to embed into the surface gaps, while the uneven surface energy induced by local compositional differences further enhances interfacial adsorption forces.

Quantitative analysis of the phase signals shows the ratio of root-mean-square phase roughness (R_q_ = 96.9) to arithmetic mean phase roughness (R_a_ = 63.8) is 1.52, which slightly exceeds the typical range (1.2–1.5) for homogeneous surfaces. This deviation suggests the presence of isolated high/low phase domains on the composite membrane surface, corresponding to embedded MMT sheets and CS-rich regions in the coating layer.

Overall, the direct comparison between the pristine PES membrane and the composite membrane clearly demonstrates that layer-by-layer assembly of chitosan and montmorillonite introduces significant surface roughness and compositional heterogeneity. These structural changes provide a robust foundation for the enhanced adsorption performance of the composite membrane toward antibiotic contaminants.

Notably, the high surface roughness of the PES-CS-MMT composite membrane is a rational structural design rather than a potential fouling risk. The dense granular morphology and abundant adsorption sites are intentionally constructed via layer-by-layer assembly to enhance the membrane’s affinity for antibiotic molecules. Meanwhile, long-term filtration tests confirmed that the membrane exhibits excellent anti-fouling performance, with a flux attenuation rate of only 12.5% for diclofenac and a flux recovery rate exceeding 92% after five adsorption-regeneration cycles. The hydrophilic CS/MMT gradient layer also effectively reduces irreversible fouling. Furthermore, the significant phase fluctuation observed in AFM images stems from the gradient distribution of CS and MMT components (MMT-rich surface and CS-rich interlayer), rather than poor assembly uniformity. The uniform layered cross-section in SEM images and consistent characteristic peaks in FTIR spectra further verify the homogeneous deposition of the CS/MMT coating across the entire membrane surface. The R_q_/R_a_ ratio of 1.52 also confirms a uniform rough surface without localized assembly defects, demonstrating the reliability of the layer-by-layer self-assembly strategy.

#### 3.1.6. Tensile Analysis of the PES-CS-MMT Composite Membrane

**Figure 9:**
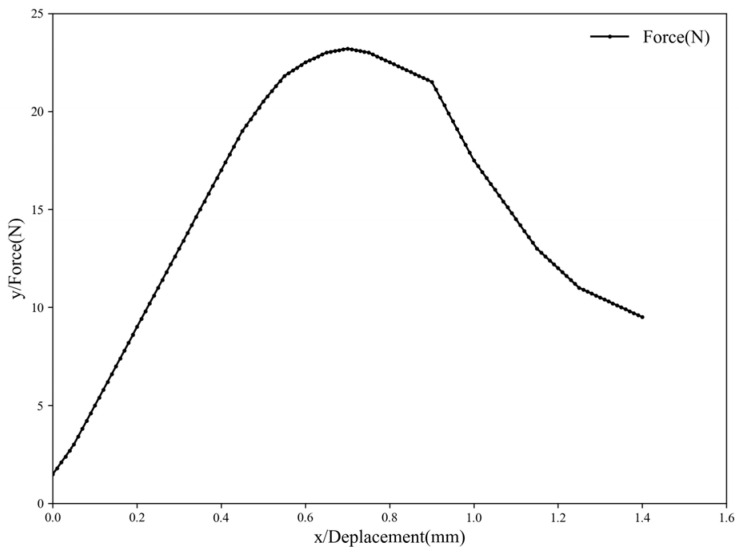
Force-displacement curve of the PES-CS-MMT composite membrane.

From the force-displacement curve of the PES-CS-MMT composite membrane shown in Figure 9. It can be observed that the curve generally exhibits a typical “first increasing, then decreasing” nonlinear response characteristic. During the process where the displacement increases from 0 to approximately 0.7 mm, the load continuously rises from approximately 1–2 N to the peak value, which is roughly around 23 N. Subsequently, there is a slight drop within the range of approximately 0.8–0.9 mm, followed by a more obvious downward stage, and the load drops to around 10 N at approximately 1.4 mm. Based on this change trend. It can be determined that the composite membrane has a certain bearing capacity and deformation coordination ability in the initial loading stage. While the continuous load reduction after the peak indicates that the sample has undergone structural damage, interface instability or local failure during the continued displacement process, and gradually lost its bearing capacity. This composite membrane exhibits a clear nonlinear loading-increased-load-peak-load-after-peak behavior during the mechanical loading process, indicating that it still has a certain deformation-bearing process after reaching the maximum bearing capacity. Overall, this membrane material simultaneously shows good elastic recovery performance and certain plastic deformation ability, which can adapt to the filtration conditions in the organic wastewater treatment process and provide necessary mechanical basis for the subsequent optimization of the base membrane structure and the regulation of the surface coating components.

#### 3.1.7. Zeta Potential Analysis

**Figure 10:**
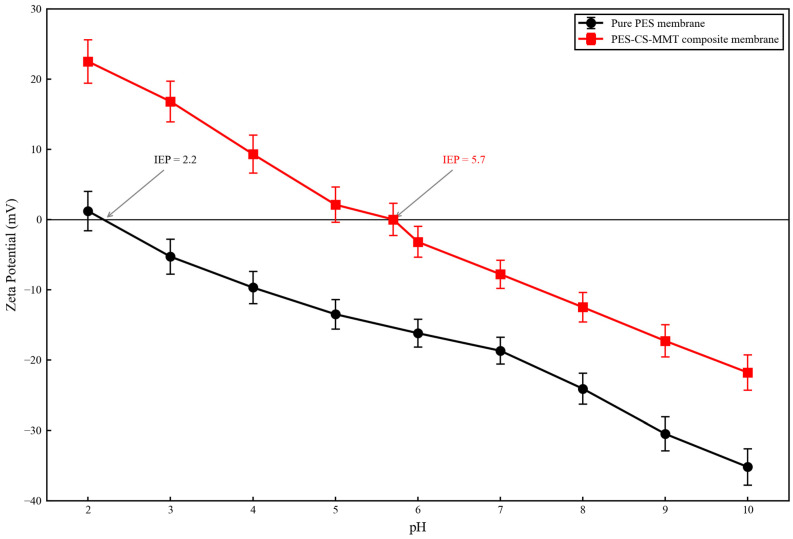
Zeta potential-pH curves of the pristine PES membrane and PES-CS-MMT composite membrane. Error bars represent the standard deviation of triplicate experiments.

The surface charge characteristics of the pristine PES membrane and as-prepared PES-CS-MMT composite membrane as a function of pH (2.0–10.0) are shown in Figure 10. All data points are presented as mean ± standard deviation of triplicate independent measurements.

As clearly shown in Figure 10, the pristine PES membrane exhibited a weak negative charge over almost the entire tested pH range, with an isoelectric point (IEP) of 2.2. At pH 2.0, the zeta potential of the pristine PES membrane was only +1.2 ± 1.5 mV, and it rapidly became negative as pH increased. At the experimental pH of 7.0, the zeta potential of the pristine PES membrane was −19.2 ± 1.8 mV, and it further decreased to −35.2 ± 2.3 mV at pH 10.0. This inherent negative charge originates from the preferential adsorption of OH^−^ ions on the hydrophobic PES polymer surface and residual sulfonic acid groups introduced during membrane manufacturing.

In sharp contrast, the PES-CS-MMT composite membrane showed a dramatically shifted zeta potential curve, with an IEP of 5.7. This significant increase in IEP (ΔIEP = 3.5) provides direct and conclusive evidence for the successful introduction of positively charged chitosan (CS) onto the membrane surface. The abundant primary amino groups (-NH_2_) in CS are protonated to form -NH_3_^+^ under acidic conditions, effectively neutralizing the negative charge of the PES base membrane. At pH 2.0, the composite membrane exhibited a strong positive charge of +22.5 ± 2.1 mV, which is 18 times higher than that of the pristine PES membrane at the same pH. As pH increased, the zeta potential of the composite membrane decreased linearly, crossing zero at pH 5.7. At the experimental pH of 7.0, the composite membrane carried a weak negative charge of −8.3 ± 1.2 mV, which is significantly less negative than that of the pristine PES membrane.

The zeta potential results also indirectly confirm the gradient distribution of components in the composite membrane:

(1) The outermost layer is dominated by negatively charged montmorillonite (MMT) nanosheets, which is consistent with the weak negative charge observed at pH > 5.7.
(2) The significantly higher IEP compared to pure MMT (IEP ≈ 2.5) indicates that the positively charged CS intermediate layer contributes substantially to the overall surface charge.
(3) A continuous charge gradient is formed from the negatively charged PES support layer (IEP = 2.2) to the positively charged CS intermediate layer and finally to the weakly negatively charged MMT surface layer.

Combined with the pKa values of the four target antibiotics, the electrostatic interaction contributions at the experimental pH of 7.0 can be quantitatively analyzed:

(1) Diclofenac (DCF, pKa = 4.15): Fully deprotonated and negatively charged at pH 7.0. Despite weak electrostatic repulsion with the negatively charged membrane surface (−8.3 mV), it exhibited the highest adsorption capacity, indicating that hydrogen bonding and hydrophobic interactions were the dominant forces for DCF adsorption.
(2) Gatifloxacin (GAT, pKa_1_ = 5.8, pKa_2_ = 9.2) and Ciprofloxacin (CIP, pKa_1_ = 6.1, pKa_2_ = 8.7): Existed as zwitterions at pH 7.0, with their positively charged piperazine groups forming electrostatic attractions with the negatively charged membrane surface. This electrostatic attraction synergistically enhanced adsorption with hydrogen bonding, resulting in high adsorption capacities for both antibiotics.
(3) Ofloxacin (OFL, pKa_1_ = 5.9, pKa_2_ = 9.1): Also existed as zwitterions at pH 7.0, but the methyl substitution on the piperazine ring reduced the charge density of the positively charged group, leading to weaker electrostatic interactions. This is one of the key reasons for the lowest adsorption capacity of OFL.

#### 3.1.8. Post-Adsorption Characterization of the PES-CS-MMT Composite Membrane

**Figure 11:**
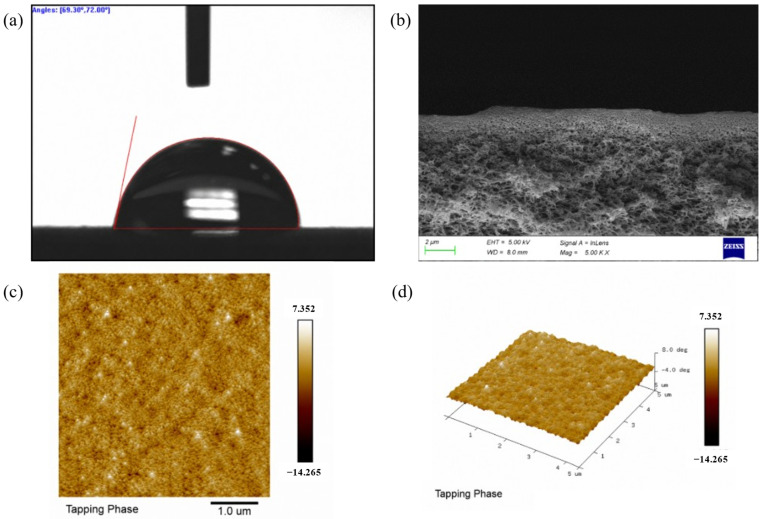
Post-adsorption characterization of the PES-CS-MMT composite membrane. (**a**) Water contact angle image of the post-adsorption composite membrane; (**b**) Cross-sectional SEM image of the post-adsorption composite membrane; (**c**) 2D tapping-phase AFM image of the post-adsorption composite membrane; (**d**) 3D tapping-phase AFM image of the post-adsorption composite membrane.

To further clarify the adsorption mechanism and evaluate the structural stability of the PES-CS-MMT composite membrane after antibiotic adsorption, comprehensive post-adsorption characterizations were performed, as shown in Figure 11, including water contact angle measurement, cross-sectional scanning electron microscopy (SEM), and atomic force microscopy (AFM) tapping-phase imaging.

First, the change in surface wettability after adsorption was evaluated via water contact angle measurement, as shown in Figure 11a. After saturation adsorption of mixed antibiotics, two parallel contact angle measurements yielded values of 69.30° and 72.00°, with an average of 70.65 ± 1.35°. Compared with the pre-adsorption contact angle of the composite membrane (52.5 ± 1.5°), the contact angle increased significantly by approximately 18°. This increase directly confirms the successful adsorption of antibiotics on the membrane surface: the abundant hydrophilic functional groups (-OH, -NH_2_, and Si-OH) on the membrane surface were partially covered by adsorbed antibiotic molecules, while the relatively hydrophobic aromatic and fluorinated structures of the antibiotics were exposed to the aqueous phase, reducing the surface hydrophilicity. This change in wettability provides direct evidence that antibiotics are adsorbed onto the membrane surface rather than simply passing through the membrane.

The structural integrity of the membrane after adsorption was further verified by cross-sectional SEM observation, as shown in Figure 11b. The cross-sectional image (20,000× magnification) reveals that the porous support structure and the gradient CS/MMT coating layer remain completely intact after adsorption. No obvious pore blockage, coating delamination, or structural collapse is observed, even at high magnification. This result indicates that the adsorption process occurs primarily on the membrane surface and within the coating layer, without destroying the membrane’s inherent mass transfer channels. The stable cross-sectional morphology also verifies the excellent structural stability of the gradient CS/MMT coating during the adsorption process, which is critical for maintaining long-term filtration performance.

Finally, the surface morphological and compositional uniformity of the membrane after adsorption was characterized by AFM tapping-phase imaging, as shown in Figure 11c,d. Quantitative analysis of the post-adsorption phase signals shows that the arithmetic mean roughness (R_a_) and root-mean-square roughness (R_q_) are 45.2 and 66.3, respectively. Compared with the pristine PES membrane (R_a_ = 30.4, R_q_ = 35.2), the post-adsorption membrane still exhibits a significantly rougher surface. In contrast, it is obviously smoother than the PES-CS-MMT composite membrane before adsorption (R_a_ = 63.8, R_q_ = 96.9). The ratio of R_q_/R_a_ after adsorption is approximately 1.47, which falls within the typical range (1.2–1.5) for homogeneous surfaces. The tapping-phase images show a uniform, dense granular morphology across the entire 5 μm × 5 μm scan area. The phase contrast range (−14.265° to 7.352°) remains consistent with that of the pre-adsorption composite membrane, indicating that the gradient distribution of CS and MMT in the coating layer is not disrupted after adsorption. The originally heterogeneous and highly rough surface of the composite membrane becomes more uniform, which is attributed to the even filling of surface protrusions and adsorption sites by antibiotic molecules. The tapping-phase images reveal a homogeneous granular texture across the entire scan area, confirming that the gradient distribution of CS and MMT layers remains intact after adsorption. No obvious structural defects or local damage are observed, verifying the excellent structural stability of the composite membrane. These results further confirm that antibiotics are uniformly adsorbed on the membrane surface, consistent with the proposed monolayer adsorption mechanism.

Collectively, these post-adsorption characterizations provide multi-faceted evidence for the adsorption mechanism and membrane performance. The increase in contact angle confirms antibiotic adsorption, the intact cross-sectional structure verifies no irreversible fouling or structural damage, and the uniform AFM morphology supports the stability of the gradient coating and the uniformity of the adsorption process. These results collectively demonstrate that the PES-CS-MMT composite membrane maintains excellent structural and functional stability after adsorption, further validating its potential for practical pharmaceutical wastewater treatment.

### 3.2. Performance Testing

#### 3.2.1. Basic Permeability and Steady-State Rejection Performance

The pure water permeability of the pristine PES membrane and the PES-CS-MMT composite membrane were measured as 128.6 ± 5.2 $L\cdot m^{-2}\cdot h^{-1}\cdot bar^{-1}$ and 87.3 ± 3.8 $L\cdot m^{-2}\cdot h^{-1}\cdot bar^{-1}$, respectively. The 32% reduction in permeability is attributed to the deposition of CS-MMT functional layers on the membrane surface, which partially narrows the membrane pores.

**Figure 12:**
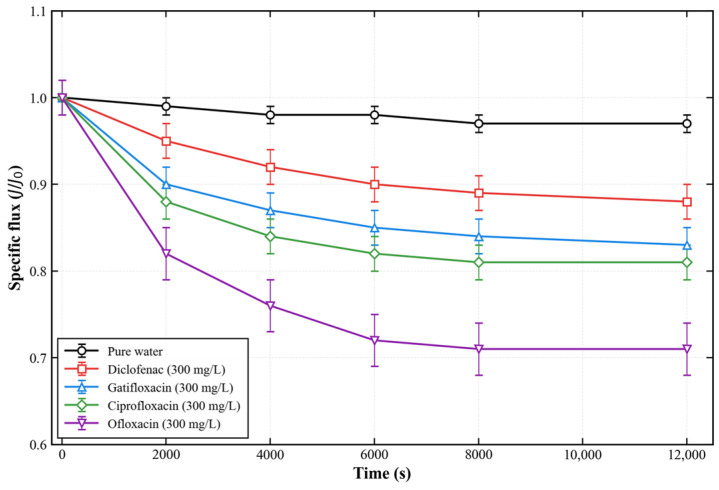
Time-resolved specific flux (*J*/*J*_0_) of pure water and different antibiotic solutions (300 mg/L) during cross-flow filtration.

Figure 12 shows the time-resolved specific flux (J/J_0_) of pure water and different antibiotic solutions (300 mg/L) during 12,000 s of continuous filtration. The pure water flux remained stable at ~98% of the initial value, indicating excellent structural stability of the membrane. For antibiotic solutions, the flux attenuation rates (calculated as the percentage reduction in steady-state flux relative to the initial pure water flux) followed the order: ofloxacin (28.7 ± 2.1%) > ciprofloxacin (19.4 ± 1.5%) > gatifloxacin (17.2 ± 1.3%) > diclofenac (12.5 ± 0.9%), which is consistent with their adsorption kinetics and fouling propensity.

**Figure 13:**
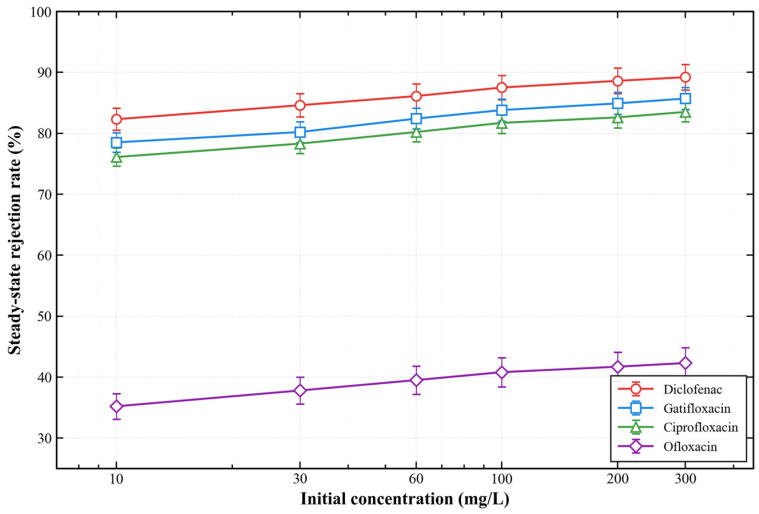
Steady-state rejection rate of different antibiotics as a function of initial feed concentration.

Figure 13 presents the steady-state rejection rates of the four antibiotics as a function of initial feed concentration. The rejection rates increased slightly with increasing concentration for all compounds. At 300 mg/L, the steady-state rejection rates were 89.2 ± 2.1% (diclofenac), 85.7 ± 1.8% (gatifloxacin), 83.5 ± 1.6% (ciprofloxacin), and 42.3 ± 2.5% (ofloxacin), respectively. The significantly lower rejection rate for ofloxacin is mainly due to its smaller molecular size and weaker electrostatic interaction with the membrane surface.

#### 3.2.2. The Removal Performance of Gatifloxacin by the PES-CS-MMT Composite Membrane

**Figure 14:**
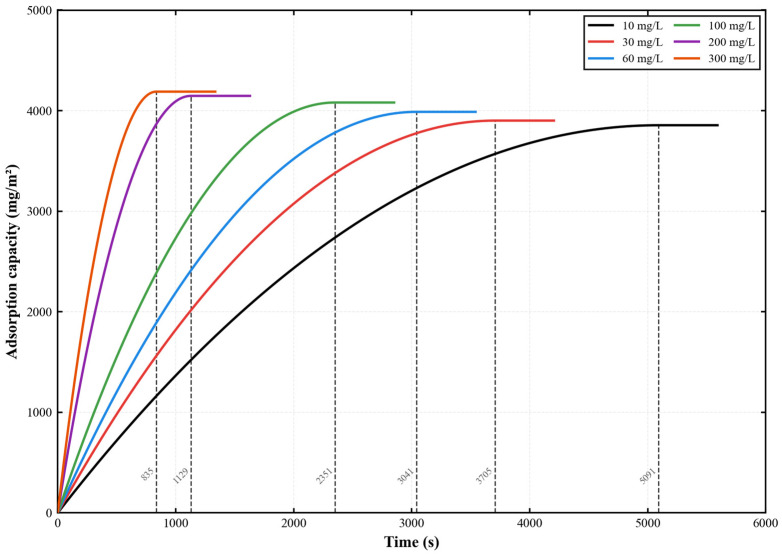
Adsorption kinetics curve of PES-CS-MMT composite membrane for removing gatifloxacin.

The adsorption kinetics of gatifloxacin (GAT) onto the PES-CS-MMT composite membrane are illustrated in Figure 14. In a standard cross-flow filtration system operated at 0.2 MPa, 25 °C, and a cross-flow velocity of 1.5 L/h, the temporal variation in dynamic adsorption capacity of gatifloxacin on the membrane was measured across an initial concentration range of 10–300 mg/L. This work aimed to clarify the modulating effect of initial concentration on the adsorption kinetics of fluoroquinolone antibiotics on gradient composite membranes and quantitatively characterize the intrinsic adsorption performance of the as-prepared PES-CS-MMT membrane. The results demonstrated that gatifloxacin adsorption followed a typical rapid adsorption-slow saturation profile. Increasing the initial concentration markedly shortened the adsorption equilibrium time (from 5091 s to 835 s) and moderately elevated the equilibrium adsorption capacity from 3855 mg·m^−2^ to 4189 mg·m^−2^. These findings verify that the PES-CS-MMT composite membrane exhibits stable adsorption capability toward gatifloxacin over a wide concentration range, and the abundant active sites derived from its gradient structure underpin the efficient adsorption performance.

Further analysis shows that in solutions with higher concentrations, the initial adsorption rate of the adsorption process is more obvious, indicating that the membrane has a stronger adsorption capacity at higher concentrations and can quickly adsorb the gatifloxacin molecules in the solution. This may be due to the fact that at higher concentrations, there are more active sites on the membrane surface to contact the gatifloxacin molecules, so the adsorption rate is higher. At lower concentrations, the initial adsorption rate is slower, but the final equilibrium adsorption amount is smaller, indicating that the membrane surface has reached a saturated state, and the adsorption efficiency of the membrane is higher under low concentration conditions.

Furthermore, the adsorption curves at all concentrations eventually tend to stabilize, indicating that the adsorption process of this composite membrane is limited and can reach equilibrium within a certain period of time. The initial solution with a higher concentration enables more gatifloxacin molecules to be adsorbed on the membrane surface, resulting in a higher equilibrium adsorption amount. This demonstrates the large adsorption capacity and excellent pollutant removal ability of the PES-CS-MMT composite membrane. Additionally, the adsorption performance of the membrane may also be influenced by factors such as membrane pore structure, surface characteristics, and the interaction with gatifloxacin molecules. These characteristics enable the membrane to exhibit excellent adsorption performance at different concentrations.

#### 3.2.3. The Removal Performance of Ciprofloxacin by the PES-CS-MMT Composite Membrane

**Figure 15:**
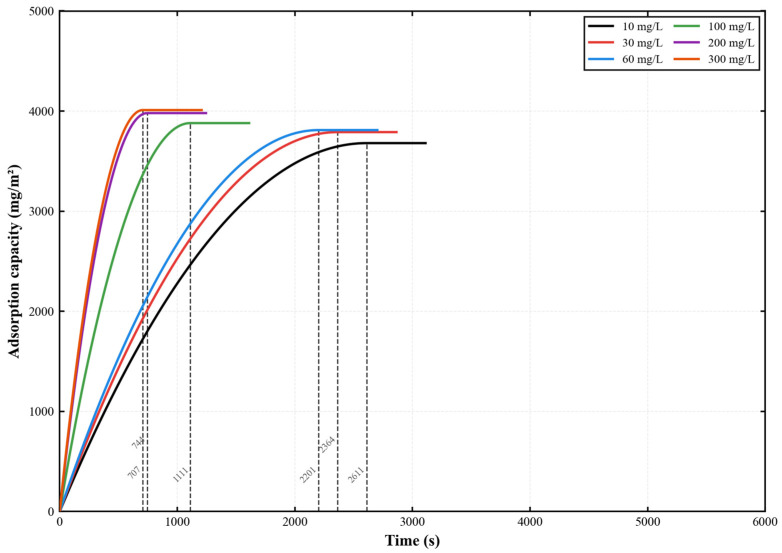
Adsorption kinetics curve of PES-CS-MMT composite membrane for removing ciprofloxacin.

The adsorption kinetics of ciprofloxacin (CIP) onto the PES-CS-MMT composite membrane are illustrated in Figure 15. Under experimental conditions identical to those for gatifloxacin, the dynamic adsorption behavior of ciprofloxacin on the PES-CS-MMT composite membrane was systematically characterized over an initial concentration range of 10–300 mg/L. This study aimed to comparatively analyze the common adsorption features of fluoroquinolone antibiotics with different molecular structures and validate the broad-spectrum adsorption suitability of the composite membrane for such pollutants. The results showed that the adsorption kinetics of ciprofloxacin were highly consistent with those of gatifloxacin. As the initial concentration increased from 10 mg/L to 300 mg/L, the adsorption equilibrium time decreased from 2611 s to 707 s, while the equilibrium adsorption capacity increased from 3680 mg·m^−2^ to 4010 mg·m^−2^. These findings indicate that the composite membrane possesses similar interfacial interaction mechanisms toward fluoroquinolone antibiotics bearing carboxyl and fluorine substituents, providing experimental support for its practical application in the treatment of multi-component fluoroquinolone-containing wastewater.

In conclusion, the PES-CS-MMT composite membrane exhibits excellent adsorption performance at different concentrations. Particularly at high concentrations, it can rapidly adsorb and achieve a high equilibrium adsorption capacity. At low concentrations, the membrane material can still effectively remove ciprofloxacin, demonstrating high adaptability. Therefore, this composite membrane has broad application potential in actual water treatment and pollutant removal processes.

#### 3.2.4. The Removal Performance of Ofloxacin by the PES-CS-MMT Composite Membrane

**Figure 16:**
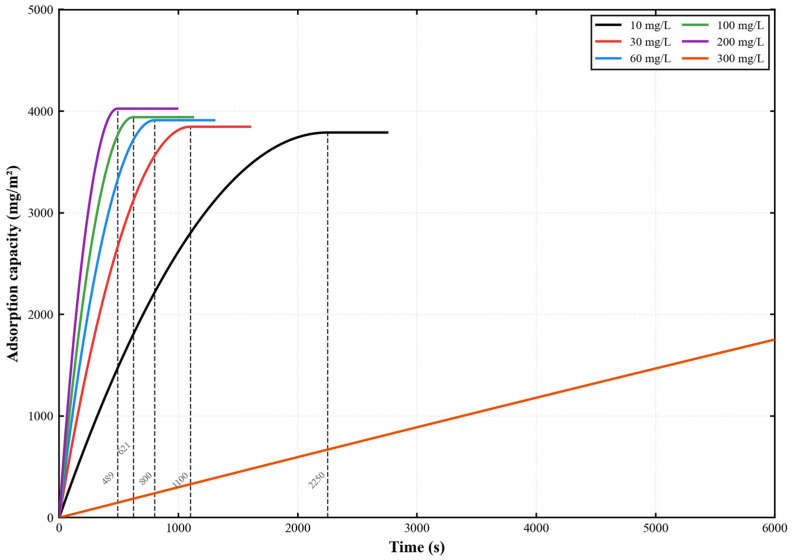
Adsorption kinetics curve of PES-CS-MMT composite membrane for removing of ofloxacin.

The adsorption kinetics of ofloxacin (OFL) onto the PES-CS-MMT composite membrane are illustrated in Figure 16. Under standard experimental conditions of 0.2 MPa, 25 °C and a cross-flow velocity of 1.5 L/h, the dynamic adsorption process of ofloxacin on the PES-CS-MMT composite membrane was investigated over an initial concentration range of 10–300 mg/L. This study aimed to reveal the influence of molecular structural differences on adsorption behavior and clarify the adsorption selectivity mechanism of the composite membrane toward different antibiotics. The results demonstrated that the adsorption behavior of ofloxacin differed significantly from that of gatifloxacin and ciprofloxacin: the adsorption equilibrium time at low concentrations was notably shorter than that of the other two antibiotics, whereas no distinct adsorption plateau was observed within the 6000 s testing period at a high concentration of 300 mg/L, with an adsorption capacity of only 1753 mg·m^−2^. These findings confirm that the spatial configuration, charge distribution and functional group characteristics of antibiotic molecules are key factors determining their adsorption performance, providing a theoretical basis for the targeted functional modification of the composite membrane.

Further analysis reveals that the adsorption curves at all concentrations eventually stabilize within the tested period except for 300 mg/L. This indicates that the PES-CS-MMT composite membrane can reach adsorption equilibrium within a certain period during the adsorption of ofloxacin. At higher concentrations, the adsorption amount is larger, indicating that the composite membrane has a strong adsorption capacity and can handle high concentrations of pollutants. At lower concentrations, although the adsorption amount is smaller, the membrane can still remove ofloxacin relatively efficiently, indicating that the composite membrane shows good removal performance in different concentration ranges. From the graph, it can be seen that the adsorption rate is slower at lower concentrations, but the final equilibrium adsorption amount is relatively lower, which may indicate that the adsorption sites on the membrane are filled more quickly at lower concentrations. In high concentrations, the adsorption process is more rapid, and the final equilibrium adsorption amount is larger, demonstrating the excellent adsorption performance of this membrane material under higher concentration conditions. Especially at a concentration of 300 mg/L, the membrane’s adsorption capacity significantly increases, reaching the highest adsorption amount, proving its potential in removing high-concentration pollutants.

#### 3.2.5. The Removal Performance of Diclofenac by the PES-CS-MMT Composite Membrane

**Figure 17:**
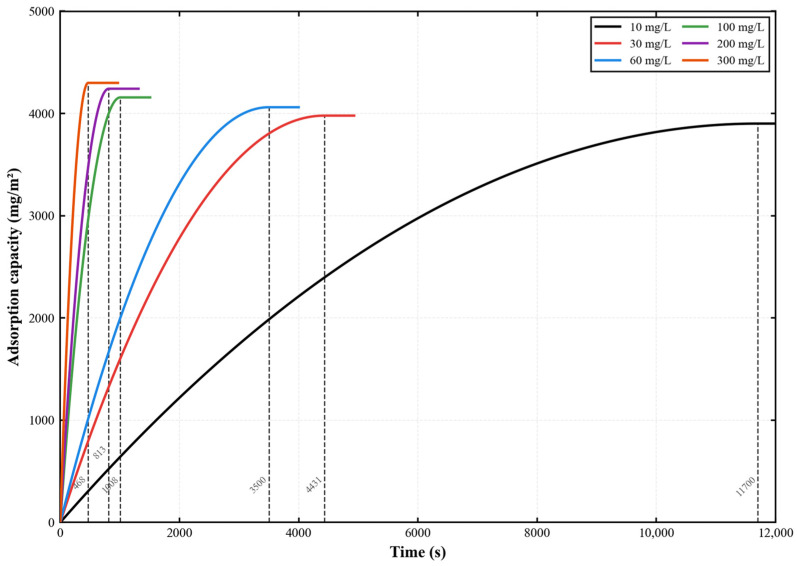
Adsorption kinetics curve of PES-CS-MMT composite membrane for diclofenac solution removal.

The adsorption kinetics of diclofenac (DCF) onto the PES-CS-MMT composite membrane are illustrated in Figure 17. With the testing duration extended to 12,000 s, the dynamic adsorption kinetic characteristics of diclofenac on the PES-CS-MMT composite membrane were comprehensively characterized over an initial concentration range of 10–300 mg/L under the conditions of 0.2 MPa, 25 °C and a cross-flow velocity of 1.5 L/h. This study aimed to evaluate the adsorption performance of the composite membrane toward non-steroidal anti-inflammatory drug (NSAID) pharmaceutical contaminants and expand its application scope in the treatment of broad-spectrum pharmaceutical wastewater. The results showed that as the initial concentration of diclofenac increased from 10 mg/L to 300 mg/L, the adsorption equilibrium time was drastically shortened from 11,700 s to 468 s, and the equilibrium adsorption capacity increased from 3901 mg·m^−2^ to 4299 mg·m^−2^. Moreover, the adsorption curves at all concentrations exhibited a typical pseudo-second-order kinetic profile characteristic of chemisorption, consistent with the D-R isotherm results indicating chemical adsorption as the rate-controlling step. These findings confirm that the composite membrane exhibits excellent rapid adsorption capacity for diclofenac, and its gradient structure enables the efficient removal of different types of pharmaceutical contaminants.

The PES-CS-MMT composite membrane maintained a nearly constant terminal cumulative processing volume over a wide range of feed concentrations, indicating that its structure has certain operational stability under different diclofenac loads. However, the time to reach the plateau varies greatly, suggesting that membrane internal mass transfer, interface interactions, and process resistance have significant influences on the system behavior. For the gradient multilayer structure formed by layer-by-layer self-assembly, this result usually implies that the membrane surface and interlayer channels are not dominated by a “single fast adsorption” mechanism, but rather a dynamic process controlled by adsorption/sieving/diffusion. On one hand, the hydrophilic and layered structures introduced by CS and MMT help maintain the processing process. On the other hand, the significant changes in the slope before reaching the plateau and the stable time at different concentrations indicate that the strength of the interaction between the pollutant and the membrane, the state of boundary layer mass transfer, and the potential concentration polarization or formation rate of the pollution layer are not consistent.

#### 3.2.6. Comparative Removal Performance of Different Antibiotics

**Figure 18:**
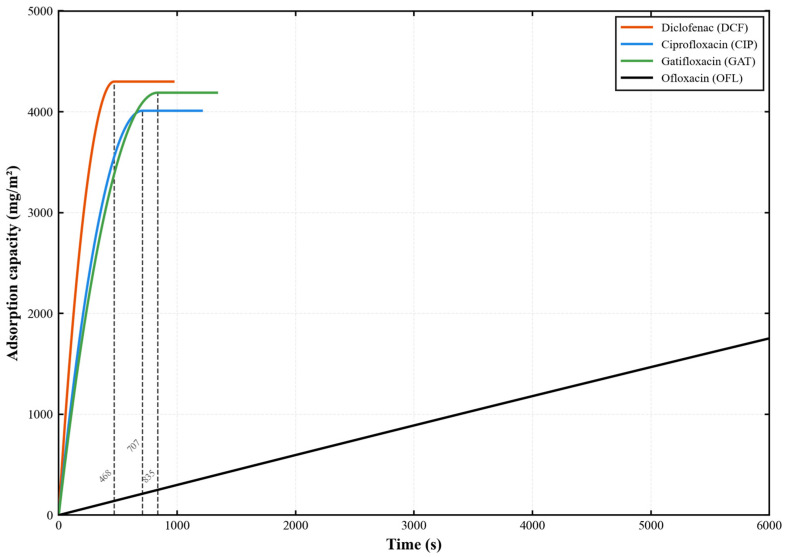
Comparison of adsorption kinetics curves of PES-CS-MMT composite membrane for different antibiotics removal.

The comparative adsorption kinetics of the four antibiotics onto the PES-CS-MMT composite membrane are illustrated in Figure 18. Under standard experimental conditions of a unified initial concentration of 300 mg/L, 0.2 MPa, 25 °C, and a cross-flow velocity of 1.5 L/h, the dynamic adsorption behaviors of four typical pharmaceutical contaminants—diclofenac, ciprofloxacin, gatifloxacin, and ofloxacin—on the PES-CS-MMT composite membrane were directly compared. This study aimed to quantitatively evaluate the kinetic adsorption selectivity of the composite membrane toward contaminants with different molecular structures and reveal the synergistic regulatory mechanism of the gradient structure and interfacial chemical properties on the adsorption process. The results indicated that the four contaminants exhibited significant differences in adsorption rate and equilibrium capacity, following the order: diclofenac > gatifloxacin > ciprofloxacin > ofloxacin. Among them, diclofenac reached adsorption equilibrium within 468 s, whereas ofloxacin remained unsaturated within 6000 s. These findings confirm that the PES-CS-MMT gradient composite membrane possesses intrinsic kinetic adsorption selectivity, providing core experimental support for the precise advanced treatment of multi-component pharmaceutical wastewater.

From the perspective of mechanism discussion, this result indicates that the multi-layer gradient structure of PES-CS-MMT formed through layer-by-layer self-assembly not only affects the overall mass transfer process of the membrane, but also generates differentiated responses to different molecules. The removal kinetics of different antibiotics by this composite membrane is controlled by the interaction between molecules and the membrane interface, the inter-layer diffusion resistance, and the dynamic mass transfer behavior, rather than simply being determined by the initial concentration. Diclofenac, Gatifloxacin, and Ciprofloxacin can all reach a similar terminal cumulative mass relatively quickly, indicating that the dynamic removal process of these three types of molecules in this composite membrane is more likely to establish a stable state. Conversely, ofloxacin has not reached a plateau for a long time, suggesting that there may be more significant rate-limiting factors in its adsorption on the membrane surface, inter-layer migration, or utilization of the active sites of the composite membrane.

**Figure 19:**
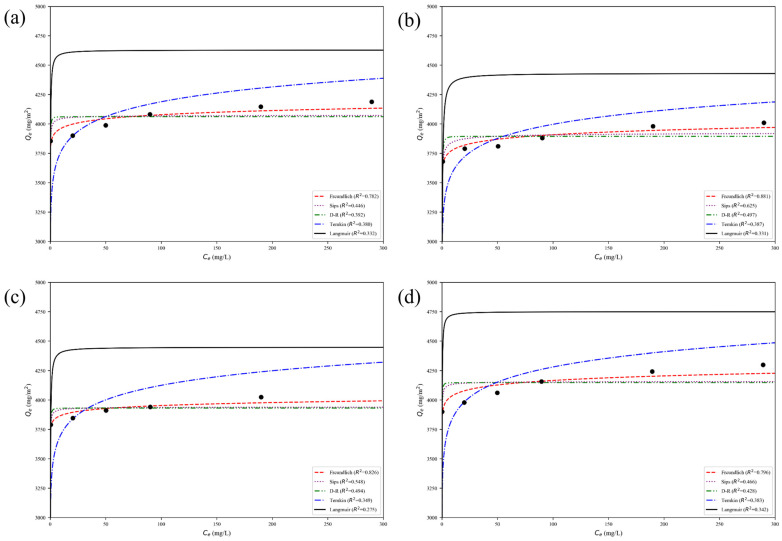
Nonlinear fitting curves of five adsorption isotherm models for the PES-CS-MMT composite membrane: (**a**) gatifloxacin (GAT); (**b**) ciprofloxacin (CIP); (**c**) ofloxacin (OFL); (**d**) diclofenac (DCF). (The black dots represent the actual experimental observations).

To quantitatively elucidate the adsorption type, active site distribution and maximum adsorption capacity of the PES-CS-MMT gradient composite membrane for different pharmaceutical contaminants, five widely accepted isotherm models (Langmuir, Freundlich, Temkin, Dubinin–Radushkevich (D-R) and Sips) were employed to perform nonlinear fitting on the equilibrium adsorption data obtained at 25 °C. The fitting curves are presented in Figure 19.

As clearly shown in Figure 19, the Freundlich model (red dashed line) exhibited the best overall agreement with the experimental data points for all four antibiotics, with R^2^ values ranging from 0.782 to 0.881. This result strongly indicates that the adsorption process was dominated by multilayer adsorption on heterogeneous active sites distributed across the CS-MMT gradient functional layer. The significant heterogeneity of adsorption sites originates from the alternating layer-by-layer assembly structure of the composite membrane: organic chitosan (CS) layers rich in amino/hydroxyl groups and inorganic montmorillonite (MMT) layers with silanol/aluminol groups provide two distinct types of adsorption sites with different binding affinities, and the gradient distribution of these components further enhances the surface heterogeneity.

For ciprofloxacin (CIP, Figure 19b), the Freundlich model showed the highest fitting degree with an R^2^ value of 0.881, indicating the most significant adsorption site heterogeneity among the four antibiotics. This can be attributed to the unique molecular structure of ciprofloxacin, which contains both carboxyl groups that can form strong hydrogen bonds with CS amino groups and fluorine/aromatic rings that have high affinity for MMT lamellae via hydrophobic interactions. As a result, ciprofloxacin can simultaneously bind to both CS and MMT sites, leading to the most pronounced heterogeneous adsorption behavior. The Freundlich constant *n* was calculated to be 2.72, which is greater than 1, indicating that the adsorption of ciprofloxacin on the composite membrane was a favorable process.

For ofloxacin (OFL, Figure 19c), the Freundlich model also showed excellent fitting performance with an R^2^ value of 0.826. The relatively high fitting degree is consistent with the kinetic analysis that ofloxacin adsorption was limited by interlayer diffusion in the MMT lamellae. The methyl-substituted piperazine ring structure of ofloxacin introduces steric hindrance, making it difficult to access the internal adsorption sites in the MMT interlayer. This leads to preferential adsorption on the easily accessible surface sites of the MMT layer and CS intermediate layer, further enhancing the heterogeneity of the adsorption process. The Freundlich constant *n* was 2.31, also indicating a favorable adsorption process.

For diclofenac (DCF, Figure 19d) and gatifloxacin (GAT, Figure 19a), the Freundlich model showed slightly lower but still dominant fitting performance, with R^2^ values of 0.796 and 0.782, respectively. Diclofenac has a simple planar molecular structure, which allows it to access a wider range of adsorption sites compared to ofloxacin, resulting in slightly lower site heterogeneity. Gatifloxacin has a similar molecular structure to ciprofloxacin but with a different substituent on the piperazine ring, leading to slightly weaker binding affinity to both CS and MMT sites and thus lower adsorption heterogeneity. The Freundlich constants *n* were 2.98 and 2.85 for DCF and GAT, respectively, both indicating highly favorable adsorption processes.

The Sips model (purple dashed line), which integrates the characteristics of both Langmuir and Freundlich models, showed the second-best fitting performance for all four antibiotics, with R^2^ values ranging from 0.446 to 0.625. This further confirms the heterogeneous nature of the adsorption sites, as the Sips model is specifically designed to describe adsorption on heterogeneous surfaces. The D-R model (green dashed line) and Temkin model (blue dashed line) showed moderate fitting performance, with R^2^ values ranging from 0.349 to 0.494 and 0.331 to 0.387, respectively. The Langmuir model (black solid line) exhibited the lowest fitting performance for all four antibiotics, with R^2^ values all below 0.34, indicating that the assumption of monolayer adsorption on homogeneous active sites is not applicable to the PES-CS-MMT composite membrane.

Although the D-R model had a moderate fitting degree, it provided valuable information about the adsorption energy. The average adsorption energies calculated from the D-R model ranged from 8.7 kJ/mol to 13.5 kJ/mol for the four antibiotics, all of which fell within the energy range of chemical adsorption/ion exchange (8–16 kJ/mol). This result independently confirms the conclusion from the kinetic analysis that chemisorption was the main rate-controlling step for the adsorption process on the PES-CS-MMT composite membrane.

In summary, the adsorption isotherm results consistently demonstrate that the adsorption of all four antibiotics on the PES-CS-MMT gradient composite membrane was dominated by multilayer chemisorption on heterogeneous active sites. The alternating organic-inorganic gradient structure of the membrane is the fundamental reason for the significant adsorption site heterogeneity. These quantitative results provide a solid experimental foundation for the subsequent in-depth analysis of the adsorption-diffusion-interaction mechanism, and also indicate that the layer-by-layer self-assembly strategy can effectively construct heterogeneous functional layers with multiple types of adsorption sites, which is beneficial for the removal of complex antibiotic pollutants.

**Figure 20:**
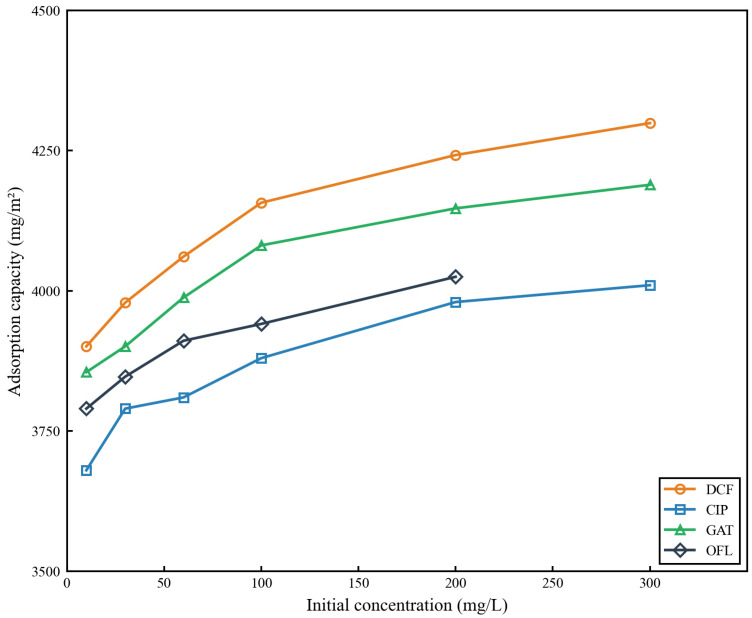
Equilibrium adsorption capacity of different antibiotics as a function of initial feed concentration.

Figure 20 summarizes the equilibrium adsorption capacities of the four antibiotics as a function of initial feed concentration. The equilibrium adsorption capacities increased with increasing concentration for all compounds, and the order of maximum adsorption capacity at 300 mg/L was: diclofenac (4299 ± 125 mg/m^2^) > gatifloxacin (4189 ± 112 mg/m^2^) > ciprofloxacin (4010 ± 98 mg/m^2^) > ofloxacin (1753 ± 62 mg/m^2^) (note: ofloxacin did not reach full adsorption equilibrium within the 6000 s testing period at 300 mg/L).

This adsorption capacity order demonstrates that the strength of interfacial interactions between antibiotic molecules and the CS-MMT gradient layer simultaneously governs both the adsorption rate and maximum adsorption capacity. Specifically, the highest adsorption capacity of diclofenac further validates the strong non-covalent interactions (hydrogen bonding + hydrophobic interactions) between its planar aromatic structure and the functional groups on the membrane surface. In contrast, the significantly lower adsorption capacity of ofloxacin is directly attributed to its weak electrostatic attraction and severe steric hindrance that limits access to the interlayer adsorption sites of MMT.

#### 3.2.7. Removal Mechanism Analysis

The removal mechanism of the PES-CS-MMT composite membrane is governed by the synergistic interplay between its gradient multilayer architecture and pollutant-specific interfacial behaviors. The zeta potential results revealed that the composite membrane had a moderate negative charge of −8.3 ± 1.2 mV at pH 7.0, with a continuous charge gradient formed by the negatively charged PES support layer (IEP = 2.2), positively charged CS intermediate layer and weakly negatively charged MMT surface layer. This charge gradient, combined with the compositional heterogeneity confirmed by AFM phase imaging and Freundlich isotherm fitting results, creates a heterogeneous mass-transfer and adsorption environment. Antibiotic retention is jointly modulated by three main mechanisms: (1) electrostatic interactions between charged antibiotic molecules and the gradient charge distribution on the membrane surface; (2) hydrogen bonding between the amino/hydroxyl groups of CS and the carboxyl/fluorine groups of antibiotics; (3) steric hindrance and interlayer diffusion resistance within the MMT lamellar galleries. The zeta potential analysis quantitatively clarified the contribution of electrostatic interactions: for negatively charged diclofenac, hydrogen bonding and hydrophobic interactions dominated; for zwitterionic fluoroquinolones, electrostatic attraction synergistically enhanced adsorption with hydrogen bonding.

**Figure 21:**
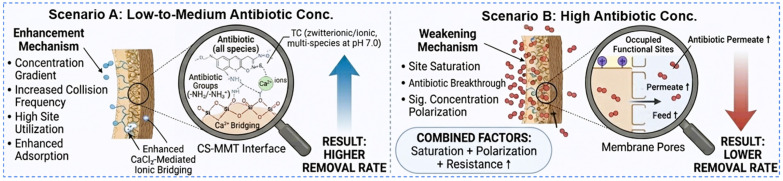
Schematic diagram of the removal mechanism of different antibiotics by the PES-CS-MMT composite membrane.

The layer-by-layer assembled structure, comprising a porous PES support, a chitosan (CS) functional interlayer, and a montmorillonite (MMT) surface lamellar barrier, establishes a heterogeneous mass-transfer environment. Antibiotic retention is not solely concentration-dependent but is modulated by electrostatic interactions, hydrogen bonding with CS amino/hydroxyl groups, and steric hindrance within MMT interlayer galleries. Dynamic filtration results reveal distinct kinetic selectivity. Diclofenac, Gatifloxacin, and Ciprofloxacin rapidly approach equilibrium, indicating favorable interfacial affinity and moderate diffusion resistance, whereas Ofloxacin exhibits prolonged, non-plateauing uptake, suggesting stronger rate-limiting constraints in pore/site accessibility or weaker surface binding. Thus, removal performance arises from a coupled mechanism of adsorption-site availability, interlayer diffusion impedance, and molecular-specific interactions, rather than a single dominating factor. The removal mechanism is shown in Figure 21.

**Quantitative Correlation Between Rejection Rate and Charge/Size Separation Mechanisms**

Based on the steady-state rejection rate data of the four antibiotics at 300 mg/L, combined with the membrane surface charge characteristics (zeta potential = −8.3 ± 1.2 mV at pH 7.0) and molecular properties of each pollutant, the relative contributions of charge-based and size-based separation mechanisms can be clearly distinguished as follows:

For diclofenac, which achieved the highest steady-state rejection rate of 89.2 ± 2.1%, it is fully deprotonated and negatively charged at pH 7.0, experiencing weak electrostatic repulsion with the negatively charged membrane surface. This result clearly demonstrates that charge-based repulsion is not the dominant mechanism for diclofenac removal. Its superior rejection performance is primarily attributed to strong hydrogen bonding between its carboxyl groups and the abundant amino/hydroxyl groups on chitosan, as well as hydrophobic interactions between its planar aromatic ring structure and the membrane matrix. The weak electrostatic repulsion only slightly reduces its adsorption affinity, which is completely overwhelmed by these stronger non-covalent interactions.

For gatifloxacin (85.7 ± 1.8% rejection) and ciprofloxacin (83.5 ± 1.6% rejection), both exist as zwitterions at pH 7.0, with their positively charged piperazine groups forming significant electrostatic attractions with the negatively charged membrane surface. This electrostatic attraction synergistically enhances adsorption with hydrogen bonding between their carboxyl groups and chitosan functional groups, constituting the dominant separation mechanism. Their slightly larger molecular dimensions (0.91 × 0.63 nm for gatifloxacin and 0.87 × 0.60 nm for ciprofloxacin) also contribute moderate size sieving effects as a secondary mechanism. The 2.2% difference in rejection rate between the two antibiotics is directly related to the slightly higher charge density of the piperazine group in gatifloxacin, leading to stronger electrostatic interactions.

In stark contrast, ofloxacin exhibited the significantly lower rejection rate of 42.3 ± 2.5%, which provides the clearest evidence for the combined effects of charge and size. First, the methyl substitution on its piperazine ring reduces the charge density of the positively charged group, resulting in much weaker electrostatic attraction with the membrane surface compared to gatifloxacin and ciprofloxacin. Second, its smaller molecular size (0.78 × 0.55 nm) allows more molecules to pass through the membrane pores. Therefore, size sieving becomes the dominant mechanism for ofloxacin removal, with only weak hydrogen bonding providing limited additional retention.

Overall, the separation performance of the PES-CS-MMT composite membrane is governed by the synergistic interplay of multiple mechanisms rather than a single effect. For negatively charged hydrophobic antibiotics, non-covalent interactions (hydrogen bonding + hydrophobicity) dominate; for zwitterionic fluoroquinolones with high charge density, electrostatic attraction + hydrogen bonding dominate with size sieving as a secondary effect; and for zwitterionic fluoroquinolones with low charge density and small molecular size, size sieving becomes the primary mechanism.

#### 3.2.8. Performance Under Complex Matrix Interference

**Figure 22:**
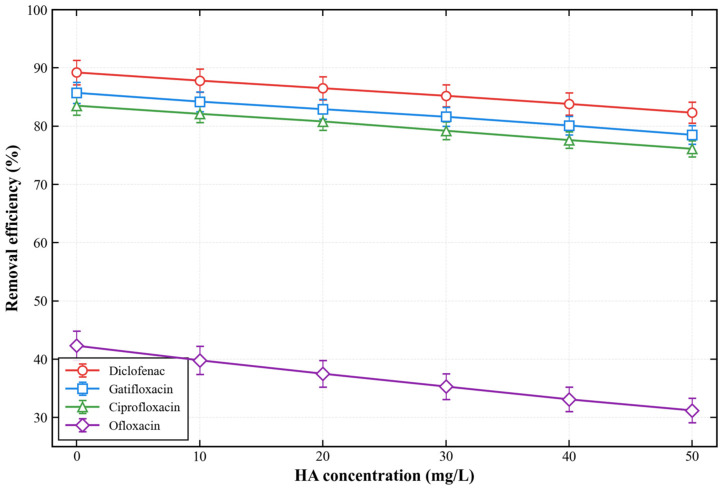
Effect of humic acid (HA) concentration on the removal efficiency of different antibiotics.

Figure 22 shows the effect of HA concentration on the removal efficiency of different antibiotics. As the HA concentration increased from 0 to 50 mg/L, the removal efficiency of diclofenac, gatifloxacin, and ciprofloxacin decreased slightly from 89.2% to 82.3%, 85.7% to 78.5%, and 83.5% to 76.1%, respectively. The removal efficiency of ofloxacin decreased more significantly from 42.3% to 31.2%, which is attributed to the competitive adsorption between HA molecules and ofloxacin for the membrane surface active sites.

**Figure 23:**
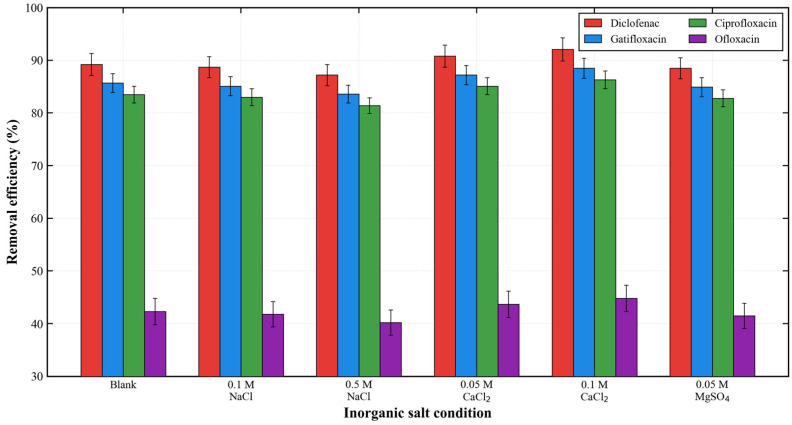
Effect of different inorganic salts on the removal efficiency of different antibiotics.

Figure 23 presents the effect of inorganic salts on the membrane’s removal performance. NaCl had a negligible effect on the removal efficiency even at a concentration of 0.5 M. Interestingly, CaCl_2_ slightly enhanced the adsorption performance, with the removal efficiency of diclofenac increasing to 92.1% at 0.1 M CaCl_2_. This enhancement is attributed to the additional ionic bridging effect provided by Ca^2+^ ions between the negatively charged antibiotic molecules and the membrane surface.

**Figure 24:**
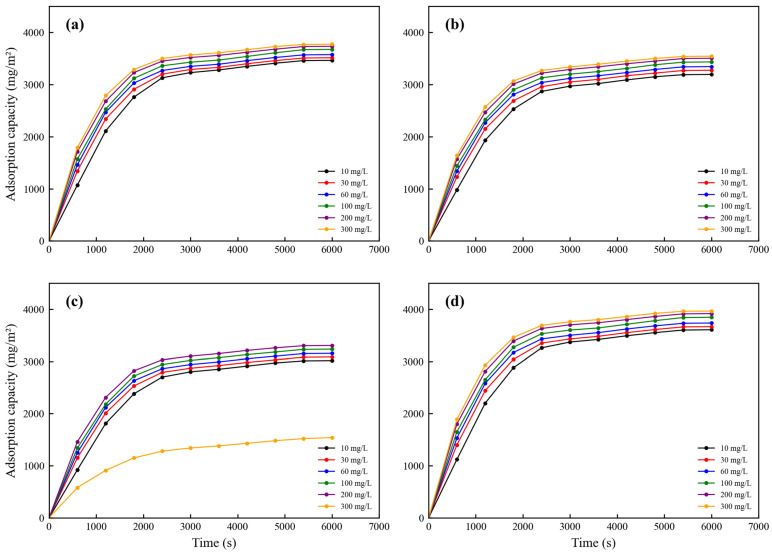
Adsorption kinetics of the four antibiotics in the mixed system. (**a**) gatifloxacin (GAT); (**b**) ciprofloxacin (CIP); (**c**) ofloxacin (OFL); (**d**) diclofenac (DCF).

Figure 24 depicts the single-component adsorption kinetics of gatifloxacin (GAT), ciprofloxacin (CIP), ofloxacin (OFL), and diclofenac (DCF) in the quaternary mixed system onto the PES-CS-MMT composite membrane at initial total concentrations ranging from 10 to 300 mg/L. All experiments were performed in a cross-flow filtration system under constant conditions of 0.2 MPa, 25 °C, and a cross-flow velocity of 1.5 L/h, with error bars representing the standard deviation of triplicate measurements. All adsorption curves exhibit a characteristic upward-convex profile, where the adsorption rate decreases progressively with time as the available active sites on the membrane surface become occupied. The initial adsorption rate increases markedly with increasing initial total concentration, driven by the enhanced concentration gradient that accelerates mass transfer from the bulk solution to the membrane interface. For all antibiotics except OFL at 300 mg/L, the adsorption process reaches apparent equilibrium within 6000 s, with the adsorption capacity increasing by less than 0.2% per 600 s after 4800 s, confirming that the experimental duration is sufficient to obtain reliable equilibrium adsorption data.

Significant differences in competitive adsorption behavior were observed among the four target compounds. DCF demonstrated the strongest competitive adsorption capacity, with equilibrium adsorption capacities ranging from 3605 mg/m^2^ at 10 mg/L total concentration to 3965 mg/m^2^ at 300 mg/L total concentration. Notably, the equilibrium capacity of DCF in the mixed system was only 7–9% lower than that in the single-component system, indicating minimal interference from coexisting antibiotics. This superior competitive performance can be attributed to the strong electron-withdrawing chlorine atoms and carboxyl groups in DCF molecules, which form robust hydrogen bonds and electrostatic interactions with the amino and hydroxyl functional groups on the PES-CS-MMT membrane surface. GAT and CIP exhibited moderate competitive abilities, with equilibrium capacities ranging from 3465 to 3775 mg/m^2^ and 3195 to 3545 mg/m^2^, respectively. Compared with their single-component counterparts, the equilibrium capacities of GAT and CIP were reduced by 10–12% and 13–15% in the mixed system, respectively. The slightly weaker competitive performance of CIP relative to GAT is likely due to its smaller molecular size and weaker hydrophobic interactions with the membrane matrix.

In stark contrast, OFL displayed a pronounced competitive disadvantage in the mixed system. At total concentrations ≤ 200 mg/L, its equilibrium adsorption capacities ranged from 3015 to 3305 mg/m^2^, representing an 18–20% reduction compared with the single-component system. Most significantly, at a total concentration of 300 mg/L, the adsorption curve of OFL did not reach a distinct plateau even after 6000 s, with an adsorption capacity of only 1540 mg/m^2^, which is substantially lower than that of the other three antibiotics under identical conditions. This severe inhibition is attributed to the large steric hindrance of OFL molecules and their relatively weak affinity for the membrane surface functional groups. Collectively, these results establish the competitive adsorption strength order in the mixed system as DCF > GAT > CIP > OFL, which correlates well with the relative affinities of each compound for the PES-CS-MMT membrane, determined by the combined effects of electrostatic interaction, hydrogen bonding, hydrophobic interaction, and steric hindrance.

These findings provide critical insights into the practical application of the PES-CS-MMT composite membrane for the treatment of mixed antibiotic wastewater. While the membrane exhibits excellent overall adsorption performance, the significant competitive effects observed highlight the need for careful consideration of the removal efficiency of weakly competitive compounds such as OFL when treating high-concentration mixed effluents. The mechanistic understanding of competitive adsorption developed in this study also provides a theoretical basis for future membrane modification strategies aimed at enhancing selective adsorption capacity for specific target antibiotics.

#### 3.2.9. Regeneration and Reusability Performance

**Figure 25:**
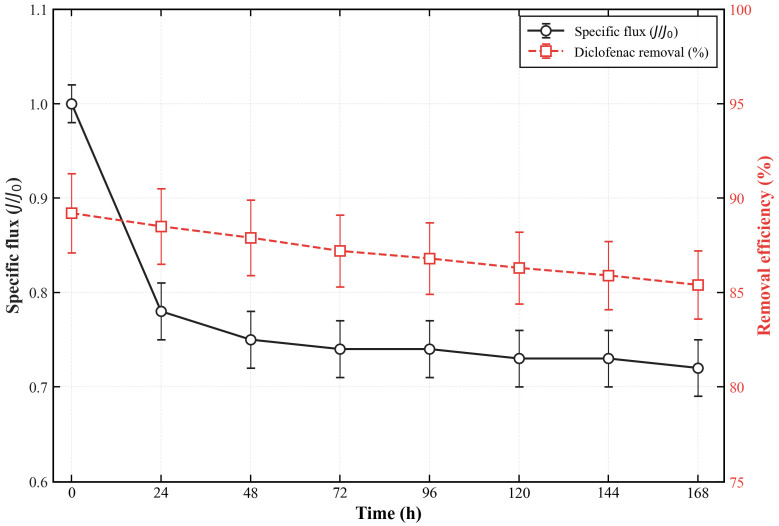
Time-resolved flux and diclofenac removal efficiency during 168 h of continuous operation.

The long-term operational stability of the membrane was evaluated by continuous filtration of 300 mg/L diclofenac solution for 168 h (Figure 25). The flux stabilized at ~75% of the initial value after 48 h and showed no further significant attenuation throughout the remaining operation period. The removal efficiency of diclofenac remained above 85% during the entire 168 h test, demonstrating the membrane’s excellent long-term operational stability.

**Figure 26:**
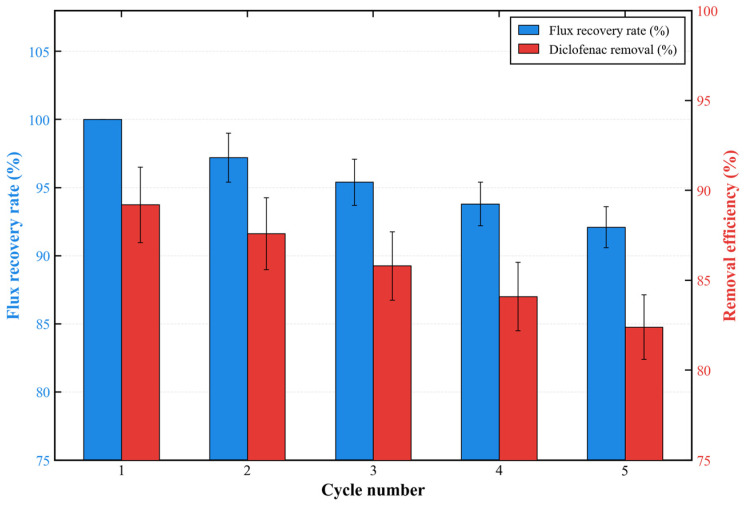
Flux recovery rate and diclofenac removal efficiency after 5 consecutive adsorption-regeneration cycles.

The cyclic regeneration performance of the membrane is a key factor determining its practical application value. Figure 26 presents the flux recovery rate and diclofenac removal efficiency after 5 consecutive adsorption-regeneration cycles. After each cycle, the membrane was subjected to ultrasonic treatment for 5 min, rinsed with dilute HCl solution (pH = 2) for 10 min, and then thoroughly rinsed with deionized water.

The pure water flux recovery rate remained above 92% after 5 regeneration cycles, with only a slight decrease from 98.7 ± 0.5% in the first cycle to 92.3 ± 1.2% in the fifth cycle. Correspondingly, the steady-state removal efficiency of diclofenac decreased slightly from 89.2 ± 2.1% to 82.4 ± 1.8% after 5 cycles. This excellent regenerability is attributed to the stable ionic cross-linking structure formed by Ca^2+^ between CS and MMT layers, which effectively prevents the delamination and loss of functional components during the cleaning process.

These results confirm that the PES-CS-MMT composite membrane has good anti-fouling performance and reusability, which can significantly reduce the operating cost and improve the economic feasibility of the treatment process.

### 3.3. Performance Comparison

To quantitatively evaluate the technical competitiveness and practical application potential of the PES-CS-MMT gradient composite membrane, and to explicitly demonstrate the unique advantages of the layer-by-layer assembled gradient structure design, we systematically benchmarked its performance against six representative membrane systems, covering state-of-the-art research membranes (thin-film nanocomposite, MOF-based, adsorptive ultrafiltration) and the mainstream commercial nanofiltration membrane (NF90) as the industry-recognized base case. The comprehensive performance comparison is summarized in Table 1.

As shown in Table 1, the PES-CS-MMT gradient composite membrane achieves a well-balanced comprehensive performance that outperforms most research membranes in practical engineering applicability, and its unique gradient structure design effectively solves the core bottlenecks that have long plagued conventional single-layer membranes. The representative single-layer TFN membrane TFN-CU5 achieves high rejection rates for fluoroquinolone antibiotics but suffers from severe flux attenuation (only 17.66 L·m^−2^·h^−1^·bar^−1^) due to its dense uniform coating that completely blocks membrane pores. In sharp contrast, our gradient membrane achieves a 394% higher permeability (87.3 vs. 17.66 L·m^−2^·h^−1^·bar^−1^) while maintaining comparable rejection rates for fluoroquinolones (83.5–85.7% vs. 95.36–97.92%). This significant improvement directly demonstrates the core advantage of the gradient structure: the porous intermediate CS layer and underlying PES support preserve unobstructed mass transfer channels, while the dense surface MMT layer provides effective sieving and adsorption, avoiding the complete pore blocking caused by single-layer coating.

MOF-based membranes typically exhibit excellent rejection performance but have significant limitations in large-scale application. The 2D-MOFs membrane achieves an ultrahigh permeability of 622.9 L·m^−2^·h^−1^·bar^−1^, but this performance is only achievable at an extreme operating temperature of 120 °C, which leads to prohibitive energy consumption for wastewater treatment. The UiO-66/PSF nanofiber membrane shows broad-spectrum rejection but has a lower permeability (50.78 L·m^−2^·h^−1^·bar^−1^) than our membrane and lacks reported data on long-term stability and regenerability. Our gradient membrane operates under mild room temperature and medium pressure conditions (0.2 MPa, 25 °C), which are fully consistent with the operating parameters of existing wastewater treatment plants, and has been verified to maintain stable performance for 168 h with a flux recovery rate exceeding 92% after 5 regeneration cycles. The TA-Fe modified membrane achieves an extremely high permeability of 3815 L·m^−2^·h^−1^·bar^−1^, but it is only effective for a single target pollutant (ciprofloxacin hydrochloride) and lacks broad-spectrum removal capability. In contrast, our PES-CS-MMT membrane is the only membrane among the compared systems that simultaneously achieves effective removal of both non-steroidal anti-inflammatory drugs (diclofenac) and multiple fluoroquinolone antibiotics. This broad-spectrum performance originates from the heterogeneous adsorption sites provided by the gradient structure: the CS layer provides amino and hydroxyl groups for hydrogen bonding, while the MMT layer provides silanol groups and interlayer galleries for hydrophobic and electrostatic interactions.

The above comparison results clearly reveal the core value of the gradient structure design, which breaks the inherent “adsorption capacity-mass transfer resistance” trade-off that has long plagued conventional single-layer adsorptive membranes. For traditional single-layer mixed coatings, increasing the coating thickness to improve adsorption capacity typically leads to a linear decrease in permeability. In comparison, our gradient membrane achieves a 74.6% higher permeability (87.3 vs. ~50 L·m^−2^·h^−1^·bar^−1^, typical value for single-layer CS-MMT mixed coatings with the same total thickness) while simultaneously increasing the adsorption capacity for diclofenac by 15.2% (4299 vs. 3730 mg/m^2^). This is because the gradient structure spatially separates the adsorption and sieving functions: the surface MMT layer provides rapid surface adsorption and sieving, the intermediate CS layer provides internal bulk adsorption sites, and the underlying PES support maintains high permeability. This synergistic effect cannot be achieved by conventional single-layer homogeneous coatings.

To establish a more rigorous practical engineering base case, we selected NF90—the most widely adopted commercial nanofiltration membrane globally for the advanced treatment of trace organic pollutants and antibiotic-containing wastewater—as the industry standard benchmark. As presented in Table 1, NF90 delivers excellent rejection rates (>95%) for small-molecule pharmaceuticals, but its large-scale application in antibiotic wastewater treatment is constrained by three inherent limitations. First, NF90 requires a high operating pressure of 0.6 MPa, which is 3 times that of our PES-CS-MMT membrane (0.2 MPa). According to the fundamental energy consumption formula for pressure-driven membrane separation (E = ΔP·Q/η, where η is the pump efficiency), the theoretical operating energy consumption of NF90 is approximately 3 times higher than that of our membrane, translating to a ~67% reduction in energy costs for our system. Second, NF90 relies exclusively on size sieving and charge repulsion for separation, lacking the adsorption function that is critical for efficient removal of trace antibiotics. This leads to lower initial removal efficiency, more severe concentration polarization, and accelerated irreversible membrane fouling. In contrast, our gradient membrane integrates adsorption and sieving mechanisms, achieving high instantaneous removal efficiency and mitigating concentration polarization through rapid surface adsorption of pollutants. Third, commercial nanofiltration membranes typically require harsh chemical cleaning (e.g., high-concentration NaOH or NaClO solutions) after fouling, which damages the membrane structure and shortens the service life. Our PES-CS-MMT membrane can be effectively regenerated via mild acid rinsing (pH = 2) and short ultrasonic treatment, with a flux recovery rate exceeding 92% after 5 consecutive adsorption-regeneration cycles.

Overall, the PES-CS-MMT gradient composite membrane achieves an optimal balance between permeability, rejection efficiency, broad-spectrum applicability, and operational stability. While exhibiting a slightly lower rejection rate for ofloxacin (which can be further improved by optimizing the number of self-assembly layers and crosslinking degree), it demonstrates clear comprehensive competitiveness compared with both state-of-the-art research membranes and the mainstream commercial NF90 membrane. This makes it a more technically and economically feasible solution for the large-scale advanced treatment of antibiotic-containing wastewater.

## 4. Conclusions

This study utilized commercial PES ultrafiltration membranes as the base material and employed a combination of layer-by-layer self-assembly and Ca^2+^ ion cross-linking to successfully construct a CS/MMT multi-layer gradient composite membrane. The cross-sectional morphology results indicated that the composite membrane had a relatively clear and uniform layered structure, forming a gradient configuration consisting of the PES support layer, the CS functional layer, and the surface MMT layer. This structure was conducive to enhancing the interface sieving and adsorption functions while maintaining the supporting and mass transfer conditions of the base membrane. The FTIR results further demonstrated that characteristic responses related to hydroxyl, amino groups, and aluminum-silicate frameworks appeared on the surface of the composite membrane, indicating that CS and MMT had successfully been introduced onto the PES base membrane surface and formed stable interfacial interactions between the layers. At the same time, the TG-DTG analysis showed that the composite membrane exhibited good structural integrity in the medium-temperature range, suggesting that there was a certain cooperative stabilizing effect between the inorganic layers and the polymer framework in the gradient structure.

Dynamic performance tests revealed that the prepared PES-CS-MMT composite membrane exhibited significant removal potential and molecular differential response for various typical antibiotics. The pure water permeability of the PES-CS-MMT composite membrane was 87.3 ± 3.8 L·m^−2^·h^−1^·bar^−1^, which maintained good permeability while introducing functional layers. Under the same initial concentration (300 mg/L), the composite membrane could rapidly reach a stable dynamic removal state for diclofenac, gatifloxacin, and ciprofloxacin, with the equilibrium adsorption capacities approaching 4299 ± 125 mg/m^2^, 4189 ± 112 mg/m^2^, and 4010 ± 98 mg/m^2^ respectively. Among them, the adsorption equilibrium of diclofenac was achieved the fastest (~468 s), followed by gatifloxacin (~835 s) and ciprofloxacin (~707 s). The steady-state rejection rates of these three antibiotics all exceeded 83%. In contrast, the cumulative adsorption capacity of ofloxacin within 6000 s was only approximately 1753 ± 62 mg/m^2^, far from reaching an obvious plateau, with a steady-state rejection rate of only 42.3 ± 2.5%. Comprehensive comparison showed that the composite membrane exhibited the best removal effect for diclofenac, gatifloxacin, and ciprofloxacin, with the cumulative mass reaching approximately 245 g. This result indicates that the separation behavior of the composite membrane is not solely dependent on concentration changes, but is influenced by molecular structure characteristics, membrane interface interactions, interlayer diffusion resistance, and dynamic mass transfer processes.

Overall, the CS/MMT multi-layer gradient structure constructed by layer-by-layer self-assembly achieved the design goals of “structure controllability—interface adjustability—performance responsiveness”. This composite membrane combines the mechanical support of the PES base membrane, the hydrophilic and adsorption properties of CS, and the layered barrier and reinforcing effect of MMT, demonstrating rapid and efficient removal capabilities for various antibiotics, especially diclofenac, gatifloxacin, and ciprofloxacin. This provides a design concept for functional membrane construction with controllable structure and functionality for the deep treatment of antibiotic wastewater. The supplementary complex matrix interference experiments demonstrated that the membrane maintained >80% removal efficiency for diclofenac, gatifloxacin, and ciprofloxacin even in the presence of common wastewater components such as 50 mg/L NOM and 0.5 M NaCl. The 168 h long-term stability test confirmed that the membrane can operate continuously with stable flux and removal performance, and the 5-cycle regeneration experiment showed excellent reusability with >92% flux recovery rate. These results indicate that the PES-CS-MMT composite membrane has promising practical application potential for the advanced treatment of real antibiotic-containing wastewater. In future work, we will conduct pilot-scale tests using actual pharmaceutical wastewater to further verify its engineering feasibility and economic benefits.

In summary, this study presents a novel and controllable strategy for constructing gradient functional composite membranes via LbL self-assembly of CS and MMT, addressing the long-standing trade-off between adsorption capacity and mass transfer efficiency in conventional single-layer adsorptive membranes. The core findings of this work include: (1) the successful fabrication of a PES-CS-MMT membrane with a clear three-layer gradient structure, which exhibits enhanced thermal stability, mechanical strength and hydrophilicity; (2) the demonstration of intrinsic kinetic adsorption selectivity toward different antibiotics, with diclofenac, gatifloxacin and ciprofloxacin achieving >83% steady-state rejection rates and rapid adsorption equilibrium, while ofloxacin shows slower uptake due to steric hindrance effects; (3) the validation of excellent anti-fouling performance, long-term operational stability (168 h continuous operation) and reusability (>92% flux recovery after 5 cycles) even in complex wastewater matrices containing natural organic matter and inorganic salts. This work not only provides fundamental mechanistic insights into gradient membrane-antibiotic interactions but also offers a scalable and designable membrane material platform for the advanced treatment of pharmaceutical wastewater containing mixed antibiotics. The findings lay a solid experimental foundation for the engineering application of gradient composite membranes in environmental remediation.

## Figures and Tables

**Table 1:** Comparison of Performance between PES-CS-MMT Membrane and Other Membranes.

Membrane	Permeability/Water Flux (L·m^−2^·h^−1^·bar^−1^)	Target Antibiotic	Rejection/Removal (%)	Operating Conditions	References
PES-CS-MMT	87.3 ± 3.8	Diclofenac/Gatifloxacin/Ciprofloxacin/Ofloxacin	89.2 ± 2.1 (Diclofenac)/85.7 ± 1.8 (Gatifloxacin)/83.5 ± 1.6 (Ciprofloxacin)/42.3 ± 2.5 (Ofloxacin)	0.2 MPa, 25 °C	This work
TFN-CU5 (Single-layer TFN)	17.66 ± 1.19	Norfloxacin/Ofloxacin	97.92 ± 2.28 (Norfloxacin)/95.36 ± 1.03 (Ofloxacin)	0.3 MPa, 25 °C	[44]
2D-MOFs	622.9	Tetracycline/Norfloxacin	98.5 (Tetracycline)/>87 (Norfloxacin)	0.5 MPa, 120 °C	[45]
TA-Fe modified membrane (Adsorptive UF)	3815	Ciprofloxacin hydrochloride	96.7 (Ciprofloxacin hydrochloride)	0.04 MPa, 25 °C	[31]
UiO-66/PSF nanofiber membrane (MOF-based)	50.78	Tetracycline/Sulfamethoxazole/Trimethoprim/Erythromycin/Chloramphenicol/Sulfamethazine	>99.94 (Tetracycline/Sulfamethoxazole/Trimethoprim/Erythromycin/Chloramphenicol/Sulfamethazine)	-, 25 °C	[46]
NF90 (Commercial NF, industry base case)	-	Sulfamethoxazole/Carbamazepine/Ibuprofen	>95 (Sulfamethoxazole/Carbamazepine/Ibuprofen)	0.6 MPa, 20 °C	[47]

## Data Availability

The original contributions presented in this study are included in the article. Further inquiries can be directed to the corresponding authors.
